# Crystal structures and Hirshfeld surface analyses of (*E*)-1-[1-(4-*tert*-butyl­phen­yl)-2,2-di­chloro­ethen­yl]-2-phenyl­diazene, (*E*)-1-[1-(4-*tert*-butyl­phen­yl)-2,2-di­chloro­ethen­yl]-2-(4-methyl­phen­yl)diazene, (*E*)-1-[1-(4-*tert*-butyl­phen­yl)-2,2-di­chloro­ethen­yl]-2-(4-meth­oxy­phen­yl)diazene and (*E*)-1-[1-(4-*tert*-butyl­phen­yl)-2,2-di­chloro­ethen­yl]-2-(3-methyl­phen­yl)diazene

**DOI:** 10.1107/S205698902300511X

**Published:** 2023-06-13

**Authors:** Abel Maharramov, Namiq Q. Shikhaliyev, Ayten Qajar, Gulnar T. Atakishiyeva, Ayten Niyazova, Victor N. Khrustalev, Mehmet Akkurt, Sema Öztürk Yıldırım, Ajaya Bhattarai

**Affiliations:** a Organic Chemistry Department, Baku State University, Z. Khalilov str. 23, AZ 1148 Baku, Azerbaijan; b Department of Engineering and Applied Sciences, Azerbaijan State University of Economics, M. Mukhtarov 194, Baku AZ1001, Azerbaijan; c Peoples’ Friendship University of Russia (RUDN University), Miklukho-Maklay St. 6, Moscow, 117198, Russian Federation; d N. D. Zelinsky Institute of Organic Chemistry RAS, Leninsky Prosp. 47, Moscow, 119991, Russian Federation; e Department of Physics, Faculty of Sciences, Erciyes University, 38039 Kayseri, Türkiye; f Department of Physics, Faculty of Science, Eskisehir Technical University, Yunus Emre Campus 26470 Eskisehir, Türkiye; g Department of Physics, Faculty of Science, Erciyes University, 38039 Kayseri, Türkiye; h Department of Chemistry, M.M.A.M.C (Tribhuvan University), Biratnagar, Nepal; Vienna University of Technology, Austria

**Keywords:** crystal structure, azo compounds, C—H⋯π and C—Cl⋯π inter­actions, Hirshfeld surface analysis

## Abstract

The crystal structures and Hirshfeld surface analyses of four similar azo compounds are reported. (*E*)-1-[1-(4-*tert*-Butyl­phen­yl)-2,2-di­chloro­ethen­yl]-2-phenyl­diazene, C_18_H_18_Cl_2_N_2_, (**I**), and (*E*)-1-[1-(4-*tert*-butyl­phen­yl)-2,2-di­chloro­ethen­yl]-2-(4-methyl­phen­yl)diazene, C_19_H_20_Cl_2_N_2_, (**II**), crystallize in the monoclinic space group *C*2/*c* with *Z* = 8, and (*E*)-1-[1-(4-*tert*-butyl­phen­yl)-2,2-di­chloro­ethen­yl]-2-(4-meth­oxy­phen­yl)diazene, C_19_H_20_Cl_2_N_2_O, (**III**), in the monoclinic space group *P*2_1_/*c* with *Z* = 4. (*E*)-1-[1-(4-*tert*-Butyl­phen­yl)-2,2-di­chloro­ethen­yl]-2-(3-methyl­phen­yl)diazene, C_19_H_20_Cl_2_N_2_, (**IV**), crystallizes in the triclinic space group *P*  with *Z* = 4 and comprises two mol­ecules (**A** and **B**) in the asymmetric unit. In the crystal structures of (**I**) and (**II**), mol­ecules are linked by C—H⋯π and C—Cl⋯π inter­actions, forming layers parallel to (  02), while mol­ecules of (**III**) are linked by C—H⋯O contacts, C—H⋯π and C—Cl⋯π inter­actions forming layers parallel to (  02). The stability of the mol­ecular packing is ensured by van der Waals forces between these layers. In the crystal structure of (**IV**), mol­ecules are linked by C—H⋯π and C—Cl⋯π inter­actions, forming a tri-periodic network.

## Abstract 2

C—H⋯π and C—Cl⋯π inter­actions are the most important inter­molecular inter­actions in the crystal structures of the title compounds.

## 1. Chemical context

The synthesis of polyfunctional compounds and the study of their structures and properties are one of the directions in organic chemistry that have been studied in detail in recent years. In this regard, the synthesis of dihalogendi­aza­butadienes from the reaction of N-substituted hydrazones of benzaldehyde derivatives with polyhalo­methanes (CCI_4_, CBr_4_) in the presence of a CuCI catalyst (Maharramov *et al.*, 2018 ▸; Shikhaliyev *et al.*, 2019*a* ▸,*b* ▸, 2021*a* ▸,*b* ▸; Nenajdenko *et al.*, 2020 ▸, 2022 ▸), the investigation of their structural features by the RQA method (Shikhaliyev *et al.*, 2021*c* ▸,*d* ▸,*e* ▸; Atioğlu *et al.*, 2020 ▸) and the investigation of the factors affecting the direction of the reaction are distinguished by their relevance.

The presence of an attached di­aza­diene system in dihalogendi­aza­butadiene derivatives leads to their application as a new class of diazo dyes, and the reaction of heminal halogen atoms with various nucleophiles results in important compounds such as azido­triazoles, hydrozo derivatives of α-ketoethers and other nitro­gen-containing heterocyclic compounds (Shikhaliyev *et al.*, 2021*f* ▸; Tsyrenova *et al.*, 2021 ▸). 
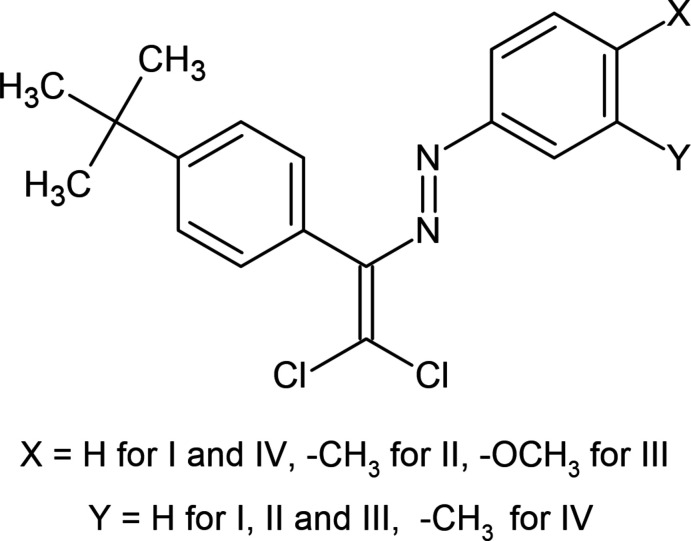


**Figure 1:**
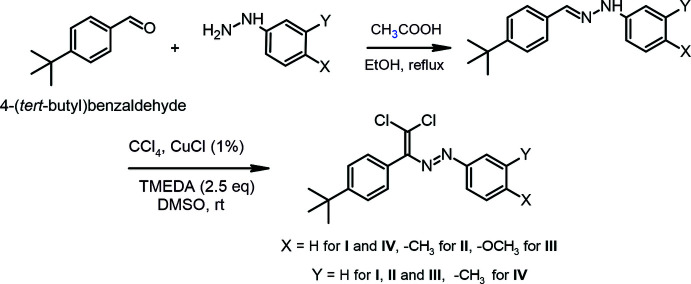
Reaction scheme for the synthesis of compounds (**I**), (**II**), (**III**) and (**IV**).

In this context, the corresponding azo dyes were synthesized based on 4-(*tert*-but­yl)benzaldehyde (Fig. 1 ▸), their crystal structures determined and their Hirshfeld surface analysed, and the results of these studies are reported in the current communication.

## 2. Structural commentary

**Figure 2:**
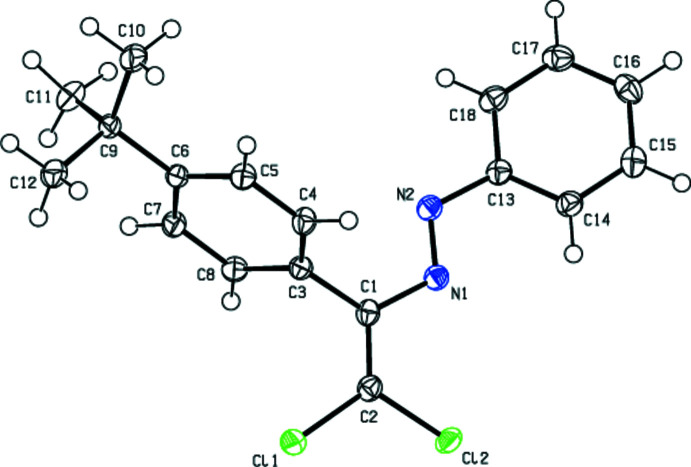
The mol­ecular structure of (**I**) with displacement ellipsoids drawn at the 50% probability level.

**Figure 3:**
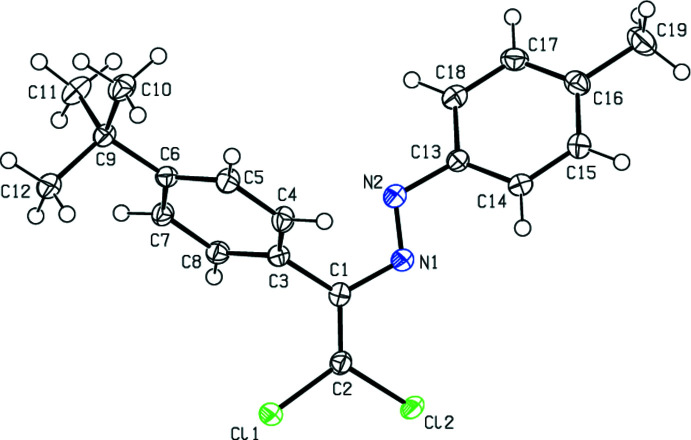
The mol­ecular structure of (**II**) with displacement ellipsoids drawn at the 50% probability level.

**Figure 4:**
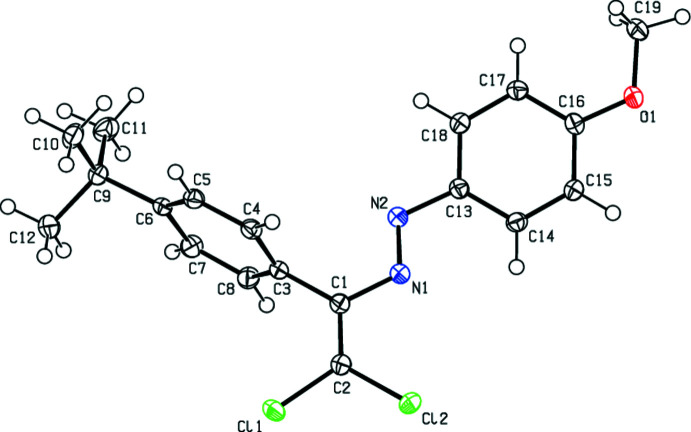
The mol­ecular structure of (**III**) with displacement ellipsoids drawn at the 50% probability level.

In the crystal structure of (**I**), the central fragment of the mol­ecule, C1/C2/N1/N2/C3/C13/Cl1/Cl2, is almost planar (Fig. 2 ▸), with an r.m.s. deviation of fitted atoms of 0.0625 Å from the least-squares plane. This plane forms a dihedral angles of 26.86 (7) and 66.71 (5)° with the planes of the phenyl (C13–C18) and 4-*tert*-butyl­phenyl (C3–C8) rings, respectively. In the crystal structure of (**II**), the central fragment (C1/C2/N2/N1/C3/C13/Cl1/Cl2; r.m.s. deviation of fitted atoms = 0.0779 Å) of the mol­ecule (Fig. 3 ▸) makes dihedral angles of 42.41 (5) and 65.31 (4)° with the planes of the 4-methyl­phenyl (C13–C18) and 4-*tert*-butyl­phenyl (C3–C8) rings, respectively. In the crystal structure of (**III**), the central fragment (C1/C2/N1/N2/C3/C13/Cl1/Cl2; r.m.s. deviation of fitted atoms = 0.0324 Å) of the mol­ecule (Fig. 4 ▸) forms dihedral angles of 10.75 (3) and 82.00 (3)° with the planes of the 4-meth­oxy­phenyl (C13–C18) and 4-*tert*-butyl­phenyl (C3–C8) rings, respectively.

**Figure 5:**
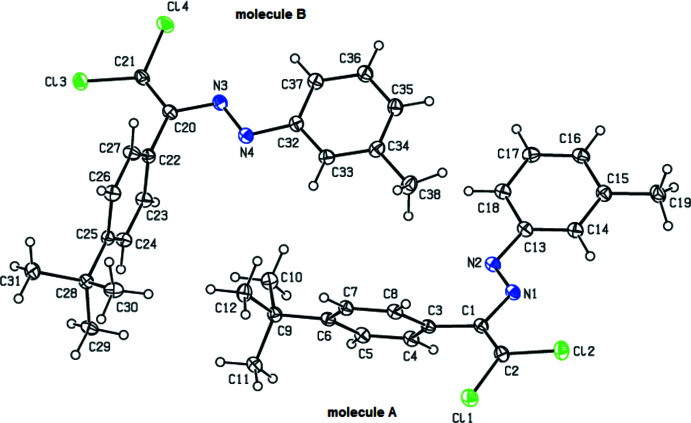
View of the two mol­ecules (**A** and **B**) in the asymmetric unit of (**IV**) with displacement ellipsoids drawn at the 30% probability level.

In the crystal structure of (**IV**), the asymmetric unit comprises two mol­ecules (**A** and **B**), Fig. 5 ▸. The central fragments (C1/C2/N1/N2/C3/C13/Cl1/Cl2 and C20/C21/N3/N4/C22/C32/Cl3/Cl4) of the mol­ecules **A** and **B** are almost planar with the r.m.s. deviations of fitted atoms being 0.0336 for **A** and 0.0243 Å for **B**. The central fragment of mol­ecule **A** forms dihedral angles of 13.45 (4) and 67.03 (5)°, respectively, with the planes of the 3-methyl­phenyl (C13–C18) and 4-*tert*-butyl­phenyl (C3–C8) rings. The central fragment of mol­ecule **B** forms dihedral angles of 3.45 (2) and 84.00 (5)°, respectively, with the planes of the 3-methyl­phenyl (C32–C37) and 4-*tert*-butyl­phenyl (C22–C27) rings.

Bond lengths and angles in all compounds are in agreement with those reported for the related azo compounds discussed in the *Database survey* section.

## 3. Supra­molecular features and Hirshfeld surface analysis

**Figure 6:**
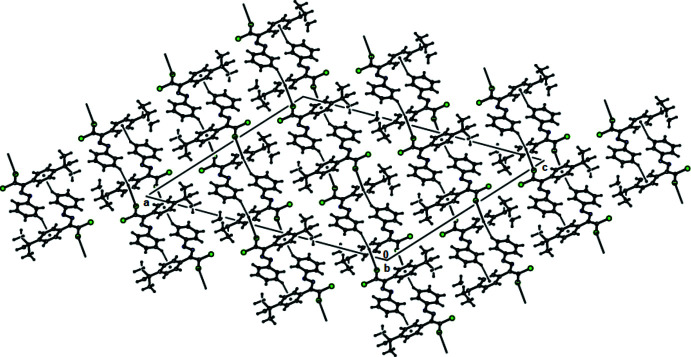
The C—Cl⋯π and C—H⋯π contacts (solid lines) of (**I**), shown along the *b* axis.

**Figure 8:**
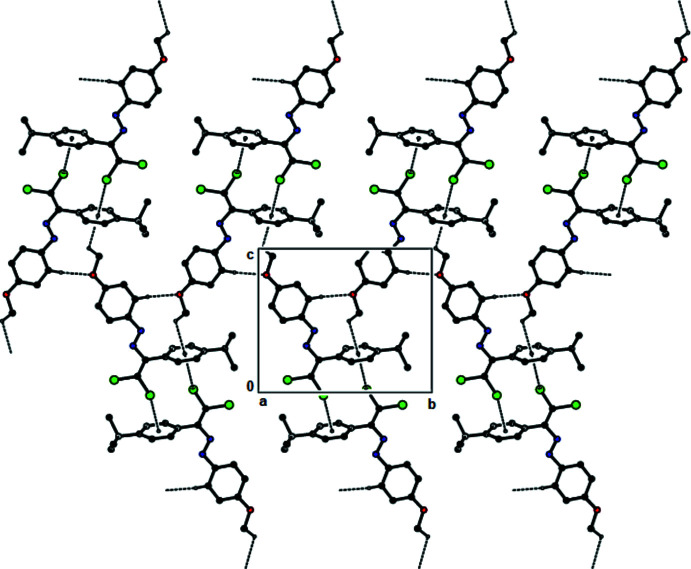
The C—H⋯O, C—Cl⋯π and C—H⋯π contacts (dashed lines) of (**III**), shown along the *a* axis.

**Figure 9:**
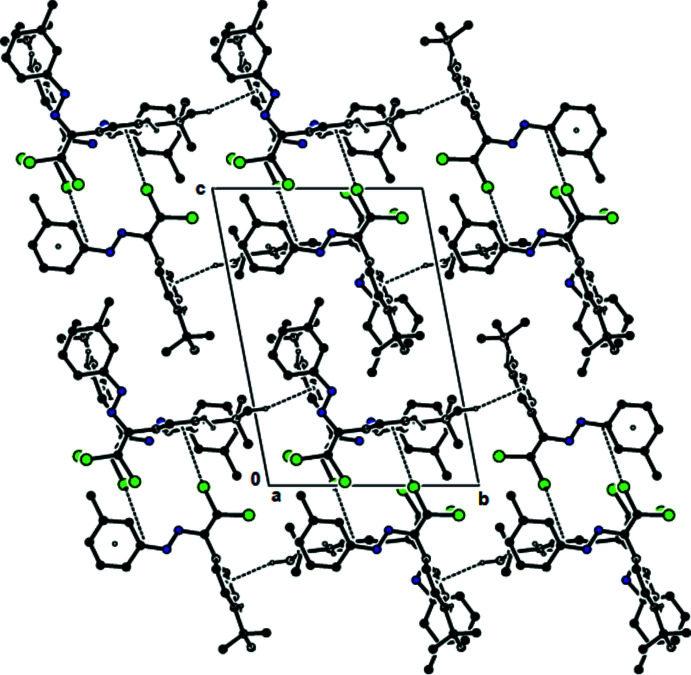
The C—Cl⋯π and C—H⋯π contacts (dashed lines) of (**IV**), shown along the *a* axis.

In the crystal structures of (**I**) and (**II**), mol­ecules are mainly connected by C—Cl⋯π inter­actions [for (**I**), C2—Cl1⋯*Cg*1^i^ = 3.5617 (8) Å; 158.39 (8)°; symmetry code: (i) 1 − *x*, −*y*, 1 − *z*, and for (**II**), C2—Cl1⋯*Cg*1^i^ = 3.6343 (1) Å; 160.79 (1)°, with *Cg*1 being the centroid of the 4-*tert*-butyl­phenyl ring (C3–C8); symmetry code: (i) 1 − *x*, −*y*, 1 − *z*]. These inter­actions, together with C—H⋯*Cg*1 inter­actions (Tables 1 ▸ and 2 ▸), lead to the formation of layers parallel to ( 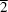 02), Figs. 6 ▸ and 7 ▸. In the crystal structure of (**III**), mol­ecules are connected by C—H⋯O and C—H⋯π inter­actions (Table 3 ▸) and additional C—Cl⋯π [C2—Cl1⋯*Cg*1^i^ = 3.7693 (1) Å; 146.35 (1) Å; *Cg*1 is the centroid of the 4-*tert*-butyl­phenyl ring (C3–C8); symmetry code: (i) 1 − *x*, −*y*, 1 − *z*], forming layers parallel to ( 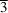 02) (Table 3 ▸, Fig. 8 ▸). van der Waals forces between these layers maintain the stability of the mol­ecular packing. In the crystal structure of (**IV**), mol­ecules are connected *via* C—H⋯π (Table 4 ▸) and C—Cl⋯π [C2—Cl2⋯*Cg*3^ii^ = 3.9515 (9) Å; C2—Cl2⋯*Cg*3^ii^ = 165.48 (1)°; symmetry code: (ii) −*x*, *y*, −1 + *z*; *Cg*3 is the centroid of the 4-*tert*-butyl­phenyl ring (C22–C27) of mol­ecule (**IVB**)] inter­actions, creating a tri-periodic network (Fig. 9 ▸).

**Figure 10:**
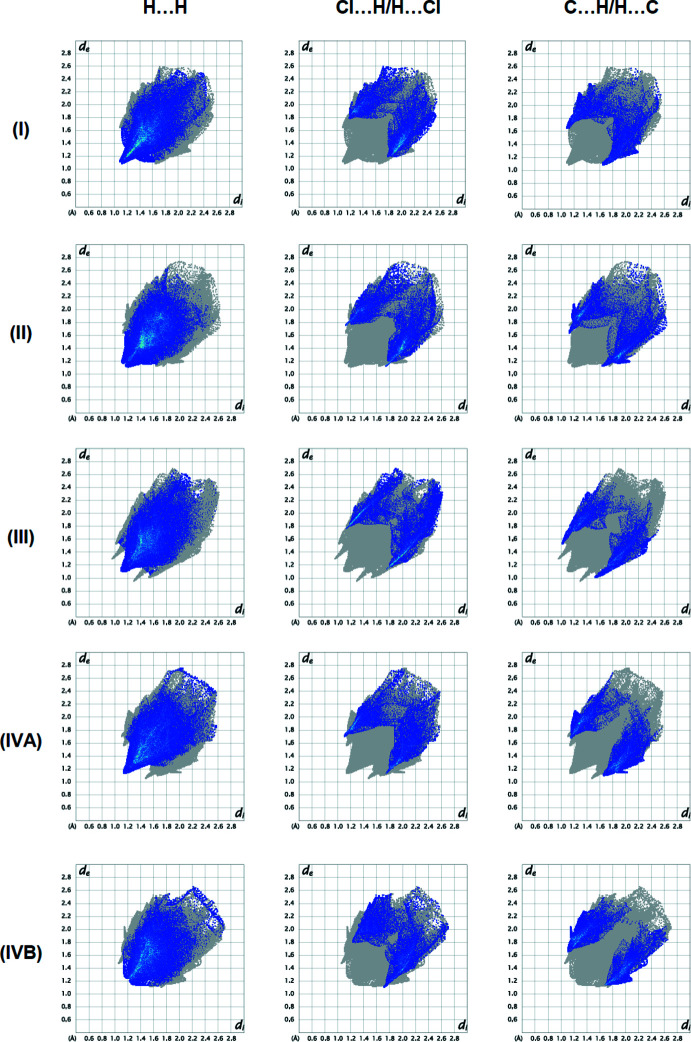
Two-dimensional fingerprint graphs showing the H⋯H, Cl⋯H/H⋯Cl and C⋯H/H⋯C inter­actions of (**I**), (**II**), (**III**), (**IVA**) and (**IV B**).

To qu­antify inter­molecular inter­actions between mol­ecules (**I**), (**II**), (**III**), (**IVA**) and (**IVB**) in their respective crystal structures, Hirshfeld surface analyses were performed, and the two-dimensional fingerprint plots generated with *CrystalExplorer17* (Spackman *et al.*, 2021 ▸). The two-dimensional fingerprint plots are shown in Fig. 10 ▸. Comparative inter­actions calculated for each compound are given in Table 5 ▸. The dominant inter­actions of all compounds are H⋯H [(**I**): 45.3%, (**II**): 47.1%, (**III**): 43.6%, (**IVA**): 47.0% and (**IVB**): 44.2%], Cl⋯H/H⋯Cl [(**I**): 22.8%, (**II**): 22.2%, (**III**): 21.3%, (**IVA**): 20.1% and (**IVB**): 19.8%] and C⋯H/H⋯C [(**I**) 17.5%, (**II**): 18.6%, (**III**): 17.0%, (**IVA**): 20.7% and (**IVB**): 21.1%]. These inter­actions play a crucial role in the overall stabilization of the crystal packing. The presence of different functional groups in the compounds leads to some differences in the remaining weak inter­actions.

## 4. Database survey

A search of the Cambridge Structural Database (CSD, Version 5.42, update of September 2021; Groom *et al.*, 2016 ▸) for the *(E)-1-(2,2-di­chloro-1-phenyl­ethen­yl)-2-phenyl­diazene* moiety resulted in 32 hits. Fourteen compounds are closely related to the title compound, *viz*. those with CSD refcodes TAZDIL (Atioğlu *et al.*, 2022*a* ▸), HEHKEO (Akkurt *et al.*, 2022 ▸), ECUDAL (Atioğlu *et al.*, 2022*b* ▸), PAXDOL (Çelikesir *et al.*, 2022 ▸), CANVUM, (Shikhaliyev *et al.*, 2021*d* ▸), EBUCUD (Shikhaliyev *et al.*, 2021*d* ▸), GUPHIL (Özkaraca *et al.*, 2020*a* ▸), DULTAI (Özkaraca *et al.*, 2020*b* ▸), XIZREG (Atioğlu *et al.*, 2019 ▸), HODQAV (Shikhaliyev *et al.*, 2019*c* ▸), HONBUK (Akkurt *et al.*, 2019 ▸), HONBOE (Akkurt *et al.*, 2019 ▸), LEQXOX (Shikhaliyev *et al.*, 2018 ▸) and LEQXIR (Shikhaliyev *et al.*, 2018 ▸).

The mol­ecules in TAZDIL are joined into layers parallel to (011) by C—H⋯O and C—H⋯F hydrogen bonds. C—Br⋯π and C—F⋯π contacts, as well as π–π stacking inter­actions strengthen the crystal packing. C—H⋯Br inter­actions connect the mol­ecules in the crystal of the polymorph-1 of HEHKEO, resulting in zigzag *C*(8) chains along [100]. These chains are connected by C—Br⋯π inter­actions into layers parallel to (001). van der Waals inter­actions between the layers contribute to the crystal cohesion. In the crystals of ECUDAL, C—H⋯O hydrogen bonds link mol­ecules into chains. These chains are linked by face-to-face π–π stacking inter­actions, resulting in a layered structure. Short inter­molecular Br⋯O contacts and van der Waals inter­actions between the layers aid in the cohesion of the crystal packing. The mol­ecules in the crystal of PAXDOL are connected into chains running parallel to [001] by C—H⋯O hydrogen bonds. C—F⋯π contacts and π–π stacking inter­actions help to consolidate the crystal packing, and short Br⋯O [2.9828 (13) Å] distances are also observed. In CANVUM, the mol­ecules are linked by C—H⋯N inter­actions along [100], forming a *C*(6) chain. The mol­ecules are further connected by C—Cl⋯π inter­actions and face-to-face π–π stacking inter­actions, resulting in ribbons along [100]. The crystal structure of EBUCUD features short C—H⋯Cl and C—H⋯O contacts and C—H⋯π and van der Waals inter­actions. In GUPHIL, mol­ecules are associated into inversion dimers *via* short Cl⋯Cl contacts [3.3763 (9) Å]. In DULTAI, the crystal structure is stabilized by a short C—H⋯Cl contact, C—Cl⋯π and van der Waals inter­actions. In XIZREG, the mol­ecules are linked by C—H⋯O hydrogen bonds into zigzag chains running along [001]. The crystal packing also features C—Cl⋯π, C—F⋯π and N—O⋯π inter­actions. In HODQAV, mol­ecules are stacked in columns along [100] *via* weak C—H⋯Cl hydrogen bonds and face-to-face π–π stacking inter­actions. The crystal packing is further consolidated by short Cl⋯Cl contacts. In HONBUK and HONBOE, mol­ecules are linked through weak *X*⋯Cl contacts (*X* = Cl for HONBUK and Br for HONBOE), C—H⋯Cl and C—Cl⋯π inter­actions into sheets parallel to (001). Additional van der Waals inter­actions consolidate the three-dimensional packing. In the crystals of LEQXOX, C—H⋯N and short Cl⋯Cl contacts are observed and in LEQXIR, C—H⋯N and C—H⋯O hydrogen bonds and short C—Cl⋯O contacts occur.

## 5. Synthesis and crystallization

Dyes (**I**), (**II**), (**III**) and (**IV**) were synthesized according to a literature protocol (Shikhaliyev *et al.*, 2018 ▸).

For (**I**), a 20 ml screw-neck vial was charged with DMSO (10 ml), (*E*)-1-(4-(*tert*-but­yl)benzyl­idene)-2-phenyl­hydrazine (252 mg, 1 mmol), tetra­methyl­ethylenedi­amine (TMEDA) (295 mg, 2.5 mmol), CuCl (2 mg, 0.02 mmol) and CBr_4_ (4.5 mmol). After 1–3 h (until TLC analysis showed complete consumption of the corresponding Schiff base), the reaction mixture was poured into a 0.01 *M* solution of HCl (100 ml, pH = 2–3), and extracted with di­chloro­methane (3× ≃ 20 ml). The combined organic phase was washed with water (3× ≃ 50 ml), brine (30 ml), dried over anhydrous Na_2_SO_4_ and concentrated *in vacuo* using a rotary evaporator. The residue was purified by column chromatography on silica gel using appropriate mixtures of hexane and di­chloro­methane (*v*/*v*: 3/1–1/1). Red solid (yield 69%); m.p. 361 K. Analysis calculated for C_18_H_18_Cl_2_N_2_ (*M* = 333.26): ^1^H NMR (300 MHz, CDCl_3_) δ 7.87 (*dd*, *J* = 6.6, 2.9 Hz, 2H), 7.54–7.47 (*m*, 5H), 7.21 (*d*, *J* = 8.3 Hz, 2H), 1.44 (*s*, 9H). ^13^C NMR (75 MHz, CDCl_3_) δ 162.3, 153.0, 152.2, 151.6, 135.1, 131.5, 129.7, 129.3, 129.0, 125.1, 123.3, 31.4, 29.8.

For (**II**), the procedure was the same as that for (**I**) using (*E*)-1-(4-(*tert*-but­yl)benzyl­idene)-2-(*p*-tol­yl)hydrazine (266 mg, 1 mmol). A red solid was obtained (yield 71%); mp 369 K. Analysis calculated for C_19_H_20_Cl_2_N_2_ (*M* = 347.28): ^1^H NMR (300 MHz, CDCl_3_) δ 7.72 (*d*, *J* = 8.3 Hz, 2H), 7.46 (*d*, *J* = 8.3 Hz, 2H), 7.25 (*d*, *J* = 8.2 Hz, 2H), 7.15 (*d*, *J* = 8.3 Hz, 2H), 2.42 (*s*, 3H), 1.39 (*s*, 9H). ^13^C NMR (75 MHz, CDCl_3_) 152.1, 151.5, 151.1, 142.2, 134.2, 129.7, 129.7, 129.4, 125.0, 123.3, 34.8, 31.3, 21.6.

For (**III**), the procedure was the same as that for (**I**) using (*E*)-1-(4-(*tert*-but­yl)benzyl­idene)-2-(4-meth­oxy­phen­yl)hydrazine (276 mg, 1 mmol). An orange solid was obtained (yield 63%); mp 400 K. Analysis calculated for C_19_H_20_Cl_2_N_2_O (*M* = 363.28): ^1^H NMR (300 MHz, CDCl_3_) δ 7.83 (*d*, *J* = 9.0 Hz, 2H), 7.48 (*d*, *J* = 8.4 Hz, 2H), 7.17 (*d*, *J* = 8.3 Hz, 2H), 6.96 (*d*, *J* = 9.0 Hz, 2H), 3.88 (*s*, 3H), 1.41 (s, 9H). ^13^C NMR (75 MHz, CDCl_3_) δ 162.5, 152.0, 151.4, 147.4, 132.9, 129.7, 129.6, 125.2, 125.0, 114.1, 55.5, 34.7, 31.3.

For (**IV**), the procedure was the same as that for (**I**) using (*E*)-1-(4-(*tert*-but­yl)benzyl­idene)-2-(m-tol­yl)hydrazine (276 mg, 1 mmol). An orange solid was obtained (yield 63%); mp 339 K. Analysis calculated for C_19_H_20_Cl_2_N_2_ (*M* = 347.28): ^1^ H NMR (300 MHz, CDCl_3_) δ 7.66 (*s*, 2H), 7.50 (*d*, *J* = 8.3 Hz, 2H), 7.37 (*dd*, *J* = 9.7, 6.0 Hz, 1H), 7.31 (*s*, 1H), 7.19 (*d*, *J* = 8.3 Hz, 2H), 2.45 (*s*, 3H), 1.43 (*s*, 9H). ^13^C NMR (75 MHz, CDCl_3_) δ δ 153.0, 152.2, 151.5, 138.9, 134.7, 132.3, 129.7, 129.3, 128.8, 125.1, 124.0, 120.3, 34.8, 31.3, 21.3.

Compounds (**I**), (**II**), (**III**) and (**IV**) were dissolved in di­chloro­methane and then left at room temperature for slow evaporation; red crystals of all compounds suitable for X-rays started to form after *ca* 2 d.

## 6. Refinement

Crystal data, data collection and structure refinement details are summarized in Table 6 ▸. For all structures, H atoms were positioned geometrically and treated as riding atoms, with C—H = 0.95–0.98 Å and *U*_iso_(H) = 1.2*U*_eq_(C) or 1.5*U*_eq_(C-meth­yl).

## Supplementary Material

Crystal structure: contains datablock(s) I, II, III, IV, global. DOI: 10.1107/S205698902300511X/wm5684sup1.cif

Structure factors: contains datablock(s) I. DOI: 10.1107/S205698902300511X/wm5684Isup2.hkl

Structure factors: contains datablock(s) II. DOI: 10.1107/S205698902300511X/wm5684IIsup3.hkl

Structure factors: contains datablock(s) III. DOI: 10.1107/S205698902300511X/wm5684IIIsup4.hkl

Structure factors: contains datablock(s) IV. DOI: 10.1107/S205698902300511X/wm5684IVsup5.hkl

CCDC references: 2268431, 2268430, 2268429, 2268428

Additional supporting information:  crystallographic information; 3D view; checkCIF report

## Figures and Tables

**Figure 7:**
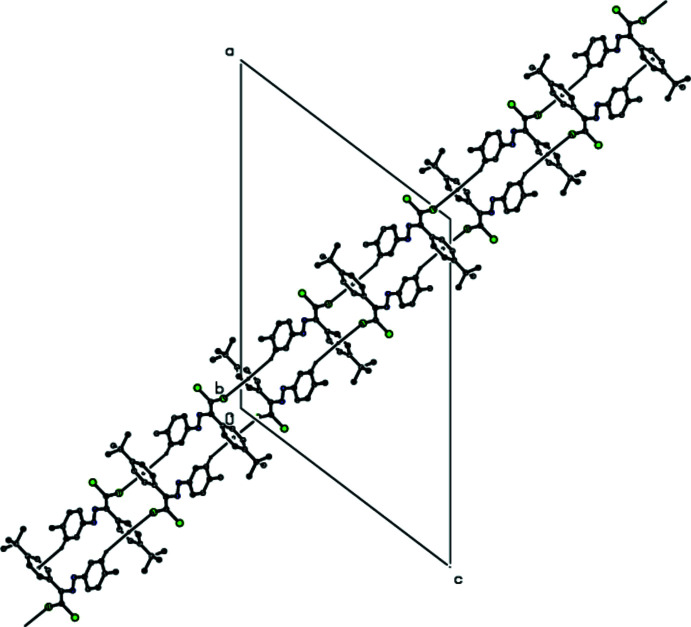
The C—Cl⋯π and C—H⋯π contacts (solid lines) of (**II**), shown along the *b* axis.

**Table 1:** Hydrogen-bond geometry (Å, °) for (**I**) *Cg*1 is the centroid of the 4-*tert*-butyl­phenyl ring (C3–C8).

*D*—H⋯*A*	*D*—H	H⋯*A*	*D*⋯*A*	*D*—H⋯*A*
C17—H17⋯*Cg*1^i^	0.95	2.95	3.476 (2)	116

Symmetry code: (i) 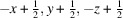 .

**Table 2:** Hydrogen-bond geometry (Å, °) for (**II**) *Cg*1 is the centroid of the 4-*tert*-butyl­phenyl ring (C3–C8).

*D*—H⋯*A*	*D*—H	H⋯*A*	*D*⋯*A*	*D*—H⋯*A*
C17—H17⋯*Cg*1^i^	0.95	2.88	3.675 (2)	142

Symmetry code: (i) 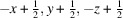 .

**Table 3:** Hydrogen-bond geometry (Å, °) for (**III**) *Cg*1 is the centroid of the 4-*tert*-butyl­phenyl ring (C3–C8).

*D*—H⋯*A*	*D*—H	H⋯*A*	*D*⋯*A*	*D*—H⋯*A*
C18—H18⋯O1^i^	0.95	2.39	3.2753 (17)	155
C19—H19*B*⋯*Cg*1^ii^	0.98	2.87	3.4276 (17)	117

Symmetry codes: (i) 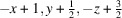 ; (ii) 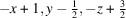 .

**Table 4:** Hydrogen-bond geometry (Å, °) for (**IV**) *Cg*1 and *Cg*2 are the centroids of the 4-*tert*-butyl­phenyl rings [(**IVA**: C3–C8 and (**IVB**): C13–C18]. *Cg*4 is the centroid of the 3-methyl­phenyl ring (C32–C37) of mol­ecule (**IVB**).

*D*—H⋯*A*	*D*—H	H⋯*A*	*D*⋯*A*	*D*—H⋯*A*
C7—H7⋯*Cg*4^i^	0.95	2.91	3.768 (2)	151
C24—H24⋯*Cg*2^ii^	0.95	2.97	3.824 (2)	150
C29—H29*B*⋯*Cg*1^iii^	0.98	2.78	3.706 (2)	157

Symmetry codes: (i) 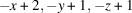 ; (ii) 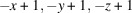 ; (iii) 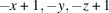 .

**Table 5:** Percentage contributions of inter­atomic contacts to the Hirshfeld surface in the crystal structure

Contact	Percentage contribution				
	(**I**)	(**II**)	(**III**)	(**IVA**)	(**IVB**)
H⋯H	45.3	47.1	43.6	47.0	44.2
Cl⋯H/H⋯Cl	22.8	22.2	21.3	20.1	19.8
C⋯H/H⋯C	17.5	18.6	17.0	20.7	21.1
N⋯H/H⋯N	5.3	5.8	3.7	7.2	8.3
O⋯H/H⋯O	–	–	5.1	–	–
Cl⋯C/C⋯Cl	3.2	2.8	2.7	2.4	3.3
C⋯C	2.4	1.2	1.7	0.3	0.3
N⋯C/C⋯N	1.5	0.7	1.4	–	–
Cl⋯N/N⋯Cl	1.2	0.5	2.9	–	–
Cl⋯Cl	0.8	1.2	0.6	2.3	3.0

**Table 6:** Experimental details

	(**I**)	(**II**)	(**III**)	(**IV**)
Crystal data				
Chemical formula	C_18_H_18_Cl_2_N_2_	C_19_H_20_Cl_2_N_2_	C_19_H_20_Cl_2_N_2_O	C_19_H_20_Cl_2_N_2_
*M*_r_	333.24	347.27	363.27	347.27
Crystal system, space group	Monoclinic, *C*2/*c*	Monoclinic, *C*2/*c*	Monoclinic, *P*2_1_/*c*	Triclinic, *P* 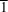
Temperature (K)	100	100	100	100
*a*, *b*, *c* (Å)	31.7847 (8), 6.0289 (1), 23.7220 (6)	30.9062 (6), 6.27248 (5), 23.3475 (4)	13.8738 (2), 12.5946 (2), 11.3013 (1)	9.8352 (2), 11.8401 (2), 16.3964 (2)
α, β, γ (°)	90, 132.669 (4), 90	90, 127.223 (3), 90	90, 112.505 (1), 90	98.397 (1), 96.189 (1), 107.149 (1)
*V* (Å^3^)	3342.4 (2)	3604.08 (15)	1824.35 (4)	1781.77 (5)
*Z*	8	8	4	4
Radiation type	Cu *K*α	Cu *K*α	Cu *K*α	Cu *K*α
μ (mm^−1^)	3.46	3.23	3.26	3.27
Crystal size (mm)	0.23 × 0.18 × 0.15	0.19 × 0.17 × 0.14	0.24 × 0.20 × 0.18	0.25 × 0.22 × 0.18

Data collection				
Diffractometer	XtaLAB Synergy, Dualflex, HyPix	XtaLAB Synergy, Dualflex, HyPix	XtaLAB Synergy, Dualflex, HyPix	XtaLAB Synergy, Dualflex, HyPix
Absorption correction	Multi-scan (*CrysAlis PRO*; Rigaku OD, 2021 ▸)	Multi-scan (*CrysAlis PRO*; Rigaku OD, 2021 ▸)	Multi-scan (*CrysAlis PRO*; Rigaku OD, 2021 ▸)	Multi-scan (*CrysAlis PRO*; Rigaku OD, 2021 ▸)
*T*_min_, *T*_max_	0.339, 0.580	0.464, 0.630	0.431, 0.550	0.328, 0.550
No. of measured, independent and observed [*I* > 2σ(*I*)] reflections	25151, 3543, 3242	28692, 3805, 3643	25894, 3843, 3603	53607, 7515, 6948
*R*_int_	0.074	0.047	0.076	0.071
(sin θ/λ)_max_ (Å^−1^)	0.634	0.633	0.634	0.634

Refinement				
*R*[*F* ^2^ > 2σ(*F* ^2^)], *wR*(*F* ^2^), *S*	0.038, 0.105, 1.08	0.033, 0.090, 1.10	0.043, 0.118, 1.06	0.058, 0.171, 1.04
No. of reflections	3543	3805	3843	7515
No. of parameters	202	213	221	423
H-atom treatment	H-atom parameters constrained	H-atom parameters constrained	H-atom parameters constrained	H-atom parameters constrained
Δρ_max_, Δρ_min_ (e Å^−3^)	0.34, −0.36	0.29, −0.30	0.53, −0.33	0.93, −0.63

Computer programs: *CrysAlis PRO* (Rigaku OD, 2021 ▸), *SHELXT2016/6* (Sheldrick, 2015*a* ▸), *SHELXL2016/6* (Sheldrick, 2015*b* ▸), *ORTEP-3 for Windows* (Farrugia, 2012 ▸) and *PLATON* (Spek, 2020 ▸).

## supplementary crystallographic information

### (E)-1-[1-(4-tert-Butylphenyl)-2,2-dichloroethenyl]-2-phenyldiazene (I) Crystal data

**Table:**

C_18_H_18_Cl_2_N_2_	*F*(000) = 1392
*M**_r_* = 333.24	*D*_x_ = 1.324 Mg m^−^^3^
Monoclinic, *C*2/*c*	Cu *K*α radiation, λ = 1.54184 Å
*a* = 31.7847 (8) Å	Cell parameters from 12999 reflections
*b* = 6.0289 (1) Å	θ = 3.7–77.3°
*c* = 23.7220 (6) Å	µ = 3.46 mm^−^^1^
β = 132.669 (4)°	*T* = 100 K
*V* = 3342.4 (2) Å^3^	Prism, red
*Z* = 8	0.23 × 0.18 × 0.15 mm

### (E)-1-[1-(4-tert-Butylphenyl)-2,2-dichloroethenyl]-2-phenyldiazene (I) Data collection

**Table:**

XtaLAB Synergy, Dualflex, HyPix diffractometer	3242 reflections with *I* > 2σ(*I*)
Radiation source: micro-focus sealed X-ray tube	*R*_int_ = 0.074
φ and ω scans	θ_max_ = 77.7°, θ_min_ = 3.7°
Absorption correction: multi-scan (*CrysAlisPro*; Rigaku OD, 2021)	*h* = −40→33
*T*_min_ = 0.339, *T*_max_ = 0.580	*k* = −7→7
25151 measured reflections	*l* = −30→30
3543 independent reflections	

### (E)-1-[1-(4-tert-Butylphenyl)-2,2-dichloroethenyl]-2-phenyldiazene (I) Refinement

**Table:**

Refinement on *F*^2^	0 restraints
Least-squares matrix: full	Hydrogen site location: inferred from neighbouring sites
*R*[*F*^2^ > 2σ(*F*^2^)] = 0.038	H-atom parameters constrained
*wR*(*F*^2^) = 0.105	*w* = 1/[σ^2^(*F*_o_^2^) + (0.0617*P*)^2^ + 1.5568*P*] where *P* = (*F*_o_^2^ + 2*F*_c_^2^)/3
*S* = 1.08	(Δ/σ)_max_ = 0.001
3543 reflections	Δρ_max_ = 0.34 e Å^−^^3^
202 parameters	Δρ_min_ = −0.35 e Å^−^^3^

### (E)-1-[1-(4-tert-Butylphenyl)-2,2-dichloroethenyl]-2-phenyldiazene (I) Special details

**Table:**

Geometry. All esds (except the esd in the dihedral angle between two l.s. planes) are estimated using the full covariance matrix. The cell esds are taken into account individually in the estimation of esds in distances, angles and torsion angles; correlations between esds in cell parameters are only used when they are defined by crystal symmetry. An approximate (isotropic) treatment of cell esds is used for estimating esds involving l.s. planes.

### (E)-1-[1-(4-tert-Butylphenyl)-2,2-dichloroethenyl]-2-phenyldiazene (I) Fractional atomic coordinates and isotropic or equivalent isotropic displacement parameters (Å^2^)

**Table:**

	*x*	*y*	*z*	*U*_iso_*/*U*_eq_	
C1	0.41113 (6)	0.3707 (3)	0.34806 (7)	0.0190 (3)	
C2	0.44632 (6)	0.2439 (3)	0.34891 (8)	0.0202 (3)	
C3	0.40396 (6)	0.3436 (2)	0.40351 (8)	0.0181 (3)	
C4	0.42175 (6)	0.5130 (3)	0.45571 (8)	0.0209 (3)	
H4	0.439678	0.641238	0.456824	0.025*	
C5	0.41349 (6)	0.4962 (2)	0.50612 (8)	0.0202 (3)	
H5	0.426029	0.613327	0.541285	0.024*	
C6	0.38719 (6)	0.3110 (2)	0.50615 (7)	0.0174 (3)	
C7	0.37085 (6)	0.1406 (3)	0.45491 (8)	0.0202 (3)	
H7	0.353829	0.010531	0.454639	0.024*	
C8	0.37887 (6)	0.1563 (3)	0.40417 (8)	0.0202 (3)	
H8	0.367082	0.037981	0.369712	0.024*	
C9	0.37725 (6)	0.2913 (2)	0.56103 (8)	0.0190 (3)	
C10	0.37583 (8)	0.5188 (3)	0.58853 (10)	0.0298 (4)	
H10A	0.413067	0.591510	0.617926	0.045*	
H10B	0.367473	0.499800	0.621133	0.045*	
H10C	0.346043	0.610581	0.544116	0.045*	
C11	0.32022 (7)	0.1755 (3)	0.52144 (9)	0.0276 (3)	
H11A	0.289387	0.251751	0.473242	0.041*	
H11B	0.312533	0.179721	0.555056	0.041*	
H11C	0.322279	0.020866	0.510718	0.041*	
C12	0.42652 (7)	0.1554 (3)	0.63116 (9)	0.0290 (4)	
H12A	0.427998	0.009439	0.614365	0.044*	
H12B	0.420195	0.136703	0.665981	0.044*	
H12C	0.462777	0.233242	0.657860	0.044*	
C13	0.31598 (6)	0.8111 (3)	0.23028 (8)	0.0190 (3)	
C14	0.33920 (6)	0.9139 (3)	0.20375 (8)	0.0223 (3)	
H14	0.373214	0.858034	0.217910	0.027*	
C15	0.31215 (7)	1.0977 (3)	0.15666 (9)	0.0263 (3)	
H15	0.327976	1.169410	0.138947	0.032*	
C16	0.26201 (7)	1.1785 (3)	0.13505 (8)	0.0258 (3)	
H16	0.243926	1.305662	0.103079	0.031*	
C17	0.23826 (6)	1.0734 (3)	0.16015 (8)	0.0260 (3)	
H17	0.203476	1.126355	0.144314	0.031*	
C18	0.26553 (6)	0.8909 (3)	0.20839 (8)	0.0231 (3)	
H18	0.249845	0.820563	0.226471	0.028*	
Cl1	0.48464 (2)	0.02700 (6)	0.41131 (2)	0.02358 (12)	
Cl2	0.45673 (2)	0.28133 (7)	0.28705 (2)	0.02385 (12)	
N1	0.38241 (5)	0.5414 (2)	0.29193 (7)	0.0193 (3)	
N2	0.34302 (5)	0.6330 (2)	0.28416 (6)	0.0199 (3)	

### (E)-1-[1-(4-tert-Butylphenyl)-2,2-dichloroethenyl]-2-phenyldiazene (I) Atomic displacement parameters (Å^2^)

**Table:**

	*U*^11^	*U*^22^	*U*^33^	*U*^12^	*U*^13^	*U*^23^
C1	0.0203 (6)	0.0199 (7)	0.0164 (6)	−0.0021 (5)	0.0123 (5)	−0.0010 (5)
C2	0.0205 (7)	0.0230 (7)	0.0170 (6)	−0.0001 (5)	0.0127 (5)	0.0002 (5)
C3	0.0195 (6)	0.0190 (7)	0.0164 (6)	0.0016 (5)	0.0123 (5)	0.0006 (5)
C4	0.0251 (7)	0.0194 (7)	0.0208 (6)	−0.0038 (5)	0.0165 (6)	−0.0018 (6)
C5	0.0245 (7)	0.0186 (7)	0.0184 (6)	−0.0035 (5)	0.0149 (6)	−0.0044 (5)
C6	0.0193 (6)	0.0179 (7)	0.0161 (6)	0.0022 (5)	0.0124 (5)	0.0014 (5)
C7	0.0245 (7)	0.0177 (7)	0.0214 (6)	−0.0027 (5)	0.0168 (6)	−0.0021 (5)
C8	0.0246 (7)	0.0179 (7)	0.0198 (6)	−0.0018 (5)	0.0157 (6)	−0.0039 (5)
C9	0.0256 (7)	0.0175 (7)	0.0190 (6)	0.0018 (5)	0.0171 (6)	0.0012 (5)
C10	0.0499 (10)	0.0213 (8)	0.0375 (8)	0.0005 (7)	0.0373 (8)	−0.0022 (7)
C11	0.0296 (8)	0.0337 (9)	0.0284 (7)	−0.0040 (7)	0.0232 (7)	−0.0027 (7)
C12	0.0323 (8)	0.0366 (9)	0.0246 (7)	0.0103 (7)	0.0218 (7)	0.0099 (7)
C13	0.0204 (6)	0.0203 (7)	0.0154 (6)	−0.0014 (5)	0.0118 (5)	−0.0025 (5)
C14	0.0238 (7)	0.0233 (7)	0.0223 (6)	0.0001 (6)	0.0167 (6)	−0.0008 (6)
C15	0.0295 (8)	0.0250 (8)	0.0261 (7)	−0.0028 (6)	0.0196 (6)	0.0012 (6)
C16	0.0282 (8)	0.0217 (8)	0.0198 (6)	0.0029 (6)	0.0133 (6)	0.0017 (6)
C17	0.0223 (7)	0.0308 (8)	0.0216 (7)	0.0048 (6)	0.0136 (6)	−0.0002 (6)
C18	0.0218 (7)	0.0291 (8)	0.0197 (6)	−0.0007 (6)	0.0145 (6)	−0.0021 (6)
Cl1	0.0259 (2)	0.0243 (2)	0.02326 (19)	0.00553 (13)	0.01777 (16)	0.00450 (13)
Cl2	0.0253 (2)	0.0312 (2)	0.02177 (19)	0.00330 (13)	0.01869 (16)	0.00240 (13)
N1	0.0210 (6)	0.0201 (6)	0.0174 (5)	−0.0001 (5)	0.0132 (5)	−0.0006 (5)
N2	0.0212 (6)	0.0217 (6)	0.0181 (5)	−0.0003 (5)	0.0138 (5)	−0.0009 (5)

### (E)-1-[1-(4-tert-Butylphenyl)-2,2-dichloroethenyl]-2-phenyldiazene (I) Geometric parameters (Å, º)

**Table:**

C1—C2	1.344 (2)	C10—H10C	0.9800
C1—N1	1.4205 (19)	C11—H11A	0.9800
C1—C3	1.4886 (19)	C11—H11B	0.9800
C2—Cl1	1.7146 (15)	C11—H11C	0.9800
C2—Cl2	1.7240 (15)	C12—H12A	0.9800
C3—C8	1.388 (2)	C12—H12B	0.9800
C3—C4	1.396 (2)	C12—H12C	0.9800
C4—C5	1.391 (2)	C13—C18	1.394 (2)
C4—H4	0.9500	C13—C14	1.398 (2)
C5—C6	1.395 (2)	C13—N2	1.4269 (19)
C5—H5	0.9500	C14—C15	1.383 (2)
C6—C7	1.396 (2)	C14—H14	0.9500
C6—C9	1.5381 (19)	C15—C16	1.390 (2)
C7—C8	1.393 (2)	C15—H15	0.9500
C7—H7	0.9500	C16—C17	1.391 (2)
C8—H8	0.9500	C16—H16	0.9500
C9—C11	1.532 (2)	C17—C18	1.388 (2)
C9—C10	1.532 (2)	C17—H17	0.9500
C9—C12	1.536 (2)	C18—H18	0.9500
C10—H10A	0.9800	N1—N2	1.2628 (18)
C10—H10B	0.9800		
			
C2—C1—N1	115.21 (13)	H10A—C10—H10C	109.5
C2—C1—C3	123.29 (13)	H10B—C10—H10C	109.5
N1—C1—C3	121.45 (13)	C9—C11—H11A	109.5
C1—C2—Cl1	122.94 (12)	C9—C11—H11B	109.5
C1—C2—Cl2	123.42 (12)	H11A—C11—H11B	109.5
Cl1—C2—Cl2	113.64 (9)	C9—C11—H11C	109.5
C8—C3—C4	118.41 (13)	H11A—C11—H11C	109.5
C8—C3—C1	122.15 (13)	H11B—C11—H11C	109.5
C4—C3—C1	119.42 (13)	C9—C12—H12A	109.5
C5—C4—C3	120.69 (14)	C9—C12—H12B	109.5
C5—C4—H4	119.7	H12A—C12—H12B	109.5
C3—C4—H4	119.7	C9—C12—H12C	109.5
C4—C5—C6	121.41 (13)	H12A—C12—H12C	109.5
C4—C5—H5	119.3	H12B—C12—H12C	109.5
C6—C5—H5	119.3	C18—C13—C14	120.28 (14)
C5—C6—C7	117.28 (13)	C18—C13—N2	115.83 (13)
C5—C6—C9	121.94 (13)	C14—C13—N2	123.75 (13)
C7—C6—C9	120.78 (13)	C15—C14—C13	119.32 (14)
C8—C7—C6	121.63 (14)	C15—C14—H14	120.3
C8—C7—H7	119.2	C13—C14—H14	120.3
C6—C7—H7	119.2	C14—C15—C16	120.54 (15)
C3—C8—C7	120.55 (13)	C14—C15—H15	119.7
C3—C8—H8	119.7	C16—C15—H15	119.7
C7—C8—H8	119.7	C15—C16—C17	120.12 (15)
C11—C9—C10	107.75 (13)	C15—C16—H16	119.9
C11—C9—C12	109.54 (13)	C17—C16—H16	119.9
C10—C9—C12	108.60 (13)	C18—C17—C16	119.81 (14)
C11—C9—C6	110.65 (12)	C18—C17—H17	120.1
C10—C9—C6	111.93 (12)	C16—C17—H17	120.1
C12—C9—C6	108.33 (12)	C17—C18—C13	119.91 (14)
C9—C10—H10A	109.5	C17—C18—H18	120.0
C9—C10—H10B	109.5	C13—C18—H18	120.0
H10A—C10—H10B	109.5	N2—N1—C1	113.43 (12)
C9—C10—H10C	109.5	N1—N2—C13	113.31 (12)
			
N1—C1—C2—Cl1	−179.36 (10)	C7—C6—C9—C11	−37.58 (18)
C3—C1—C2—Cl1	3.2 (2)	C5—C6—C9—C10	23.30 (19)
N1—C1—C2—Cl2	−0.05 (19)	C7—C6—C9—C10	−157.77 (14)
C3—C1—C2—Cl2	−177.52 (11)	C5—C6—C9—C12	−96.41 (16)
C2—C1—C3—C8	−67.0 (2)	C7—C6—C9—C12	82.52 (17)
N1—C1—C3—C8	115.67 (16)	C18—C13—C14—C15	1.1 (2)
C2—C1—C3—C4	114.42 (17)	N2—C13—C14—C15	−174.40 (14)
N1—C1—C3—C4	−62.90 (18)	C13—C14—C15—C16	−0.8 (2)
C8—C3—C4—C5	−1.2 (2)	C14—C15—C16—C17	−0.6 (2)
C1—C3—C4—C5	177.40 (13)	C15—C16—C17—C18	1.6 (2)
C3—C4—C5—C6	−0.2 (2)	C16—C17—C18—C13	−1.3 (2)
C4—C5—C6—C7	1.7 (2)	C14—C13—C18—C17	−0.1 (2)
C4—C5—C6—C9	−179.38 (13)	N2—C13—C18—C17	175.75 (13)
C5—C6—C7—C8	−1.8 (2)	C2—C1—N1—N2	169.00 (13)
C9—C6—C7—C8	179.25 (13)	C3—C1—N1—N2	−13.47 (19)
C4—C3—C8—C7	1.1 (2)	C1—N1—N2—C13	177.31 (11)
C1—C3—C8—C7	−177.48 (13)	C18—C13—N2—N1	168.30 (13)
C6—C7—C8—C3	0.4 (2)	C14—C13—N2—N1	−16.0 (2)
C5—C6—C9—C11	143.50 (14)		

### (E)-1-[1-(4-tert-Butylphenyl)-2,2-dichloroethenyl]-2-phenyldiazene (I) Hydrogen-bond geometry (Å, º)

Cg1 is the centroid of the 4-*tert*-butylphenyl ring (C3–C8).

**Table:**

*D*—H···*A*	*D*—H	H···*A*	*D*···*A*	*D*—H···*A*
C17—H17···*Cg*1^i^	0.95	2.95	3.476 (2)	116

Symmetry code: (i) −*x*+1/2, *y*+1/2, −*z*+1/2.

### (E)-1-[1-(4-tert-Butylphenyl)-2,2-dichloroethenyl]-2-\ (4-methylphenyl)diazene (II) Crystal data

**Table:**

C_19_H_20_Cl_2_N_2_	*F*(000) = 1456
*M**_r_* = 347.27	*D*_x_ = 1.280 Mg m^−^^3^
Monoclinic, *C*2/*c*	Cu *K*α radiation, λ = 1.54184 Å
*a* = 30.9062 (6) Å	Cell parameters from 19033 reflections
*b* = 6.27248 (5) Å	θ = 3.6–77.1°
*c* = 23.3475 (4) Å	µ = 3.23 mm^−^^1^
β = 127.223 (3)°	*T* = 100 K
*V* = 3604.08 (15) Å^3^	Prism, red
*Z* = 8	0.19 × 0.17 × 0.14 mm

### (E)-1-[1-(4-tert-Butylphenyl)-2,2-dichloroethenyl]-2-\ (4-methylphenyl)diazene (II) Data collection

**Table:**

XtaLAB Synergy, Dualflex, HyPix diffractometer	3643 reflections with *I* > 2σ(*I*)
Radiation source: micro-focus sealed X-ray tube	*R*_int_ = 0.047
φ and ω scans	θ_max_ = 77.5°, θ_min_ = 3.6°
Absorption correction: multi-scan (*CrysAlisPro*; Rigaku OD, 2021)	*h* = −38→38
*T*_min_ = 0.464, *T*_max_ = 0.630	*k* = −6→7
28692 measured reflections	*l* = −29→29
3805 independent reflections	

### (E)-1-[1-(4-tert-Butylphenyl)-2,2-dichloroethenyl]-2-\ (4-methylphenyl)diazene (II) Refinement

**Table:**

Refinement on *F*^2^	Hydrogen site location: inferred from neighbouring sites
Least-squares matrix: full	H-atom parameters constrained
*R*[*F*^2^ > 2σ(*F*^2^)] = 0.033	*w* = 1/[σ^2^(*F*_o_^2^) + (0.047*P*)^2^ + 2.8*P*] where *P* = (*F*_o_^2^ + 2*F*_c_^2^)/3
*wR*(*F*^2^) = 0.090	(Δ/σ)_max_ = 0.001
*S* = 1.10	Δρ_max_ = 0.29 e Å^−^^3^
3805 reflections	Δρ_min_ = −0.30 e Å^−^^3^
213 parameters	Extinction correction: *SHELXL2016/6* (Sheldrick, 2015b), Fc^*^=kFc[1+0.001xFc^2^λ^3^/sin(2θ)]^-1/4^
0 restraints	Extinction coefficient: 0.00017 (5)

### (E)-1-[1-(4-tert-Butylphenyl)-2,2-dichloroethenyl]-2-\ (4-methylphenyl)diazene (II) Special details

**Table:**

Geometry. All esds (except the esd in the dihedral angle between two l.s. planes) are estimated using the full covariance matrix. The cell esds are taken into account individually in the estimation of esds in distances, angles and torsion angles; correlations between esds in cell parameters are only used when they are defined by crystal symmetry. An approximate (isotropic) treatment of cell esds is used for estimating esds involving l.s. planes.

### (E)-1-[1-(4-tert-Butylphenyl)-2,2-dichloroethenyl]-2-\ (4-methylphenyl)diazene (II) Fractional atomic coordinates and isotropic or equivalent isotropic displacement parameters (Å^2^)

**Table:**

	*x*	*y*	*z*	*U*_iso_*/*U*_eq_	
Cl1	0.48998 (2)	0.03552 (5)	0.41749 (2)	0.02454 (11)	
Cl2	0.46363 (2)	0.25942 (5)	0.29246 (2)	0.02446 (11)	
N1	0.39101 (4)	0.51844 (18)	0.30369 (6)	0.0208 (2)	
N2	0.35039 (5)	0.59579 (18)	0.29674 (6)	0.0222 (2)	
C1	0.41805 (5)	0.3591 (2)	0.35785 (7)	0.0201 (3)	
C2	0.45276 (5)	0.2354 (2)	0.35637 (7)	0.0208 (3)	
C3	0.40920 (5)	0.3421 (2)	0.41360 (7)	0.0195 (3)	
C4	0.42254 (6)	0.5155 (2)	0.45907 (7)	0.0227 (3)	
H4	0.438159	0.639440	0.455003	0.027*	
C5	0.41316 (6)	0.5082 (2)	0.51023 (7)	0.0220 (3)	
H5	0.422523	0.627803	0.540667	0.026*	
C6	0.39028 (5)	0.3292 (2)	0.51781 (7)	0.0191 (3)	
C7	0.37776 (5)	0.1560 (2)	0.47236 (7)	0.0211 (3)	
H7	0.362819	0.030753	0.477002	0.025*	
C8	0.38657 (5)	0.1621 (2)	0.42057 (7)	0.0209 (3)	
H8	0.377088	0.042930	0.389929	0.025*	
C9	0.38015 (5)	0.3145 (2)	0.57443 (7)	0.0206 (3)	
C10	0.38607 (6)	0.5300 (2)	0.60923 (8)	0.0284 (3)	
H10A	0.423263	0.582766	0.634072	0.043*	
H10B	0.378300	0.513209	0.643923	0.043*	
H10C	0.360454	0.632136	0.571982	0.043*	
C11	0.32232 (6)	0.2333 (3)	0.53954 (8)	0.0295 (3)	
H11A	0.296101	0.325794	0.499032	0.044*	
H11B	0.315190	0.234963	0.575128	0.044*	
H11C	0.318734	0.087345	0.522185	0.044*	
C12	0.42191 (6)	0.1599 (3)	0.63358 (8)	0.0315 (3)	
H12A	0.417802	0.019311	0.612496	0.047*	
H12B	0.415934	0.147896	0.670170	0.047*	
H12C	0.458667	0.213787	0.655851	0.047*	
C13	0.32527 (5)	0.7652 (2)	0.24618 (7)	0.0199 (3)	
C14	0.35328 (5)	0.8930 (2)	0.22966 (7)	0.0221 (3)	
H14	0.390374	0.865296	0.250923	0.027*	
C15	0.32646 (6)	1.0606 (2)	0.18195 (8)	0.0252 (3)	
H15	0.345643	1.148864	0.171144	0.030*	
C16	0.27186 (6)	1.1026 (2)	0.14946 (7)	0.0247 (3)	
C17	0.24436 (6)	0.9736 (2)	0.16630 (8)	0.0263 (3)	
H17	0.206996	0.999041	0.144008	0.032*	
C18	0.27109 (6)	0.8078 (2)	0.21550 (8)	0.0261 (3)	
H18	0.252413	0.723936	0.228125	0.031*	
C19	0.24335 (7)	1.2860 (3)	0.09755 (9)	0.0361 (4)	
H19A	0.204956	1.250175	0.061231	0.054*	
H19B	0.260343	1.313112	0.073900	0.054*	
H19C	0.246248	1.413799	0.123816	0.054*	

### (E)-1-[1-(4-tert-Butylphenyl)-2,2-dichloroethenyl]-2-\ (4-methylphenyl)diazene (II) Atomic displacement parameters (Å^2^)

**Table:**

	*U*^11^	*U*^22^	*U*^33^	*U*^12^	*U*^13^	*U*^23^
Cl1	0.02633 (18)	0.02606 (18)	0.02459 (17)	0.00732 (11)	0.01716 (14)	0.00613 (11)
Cl2	0.02460 (18)	0.03214 (19)	0.02356 (17)	0.00413 (12)	0.01819 (15)	0.00397 (11)
N1	0.0216 (5)	0.0219 (5)	0.0208 (5)	0.0017 (4)	0.0139 (4)	0.0011 (4)
N2	0.0225 (5)	0.0241 (5)	0.0229 (5)	0.0014 (4)	0.0153 (5)	0.0021 (4)
C1	0.0210 (6)	0.0207 (6)	0.0197 (6)	−0.0009 (5)	0.0129 (5)	0.0008 (5)
C2	0.0207 (6)	0.0241 (6)	0.0192 (6)	0.0006 (5)	0.0129 (5)	0.0022 (5)
C3	0.0192 (6)	0.0211 (6)	0.0194 (6)	0.0021 (5)	0.0123 (5)	0.0018 (5)
C4	0.0268 (7)	0.0202 (6)	0.0242 (6)	−0.0034 (5)	0.0170 (6)	−0.0009 (5)
C5	0.0256 (6)	0.0201 (6)	0.0229 (6)	−0.0030 (5)	0.0160 (5)	−0.0029 (5)
C6	0.0178 (6)	0.0210 (6)	0.0191 (6)	0.0023 (5)	0.0115 (5)	0.0014 (5)
C7	0.0246 (6)	0.0184 (6)	0.0253 (6)	−0.0019 (5)	0.0177 (5)	−0.0005 (5)
C8	0.0236 (6)	0.0190 (6)	0.0227 (6)	−0.0001 (5)	0.0153 (5)	−0.0016 (5)
C9	0.0223 (6)	0.0225 (6)	0.0213 (6)	−0.0007 (5)	0.0154 (5)	−0.0007 (5)
C10	0.0361 (8)	0.0275 (7)	0.0312 (7)	−0.0034 (6)	0.0255 (7)	−0.0064 (6)
C11	0.0278 (7)	0.0386 (8)	0.0303 (7)	−0.0081 (6)	0.0218 (6)	−0.0078 (6)
C12	0.0361 (8)	0.0381 (8)	0.0308 (7)	0.0110 (6)	0.0258 (7)	0.0103 (6)
C13	0.0225 (6)	0.0208 (6)	0.0188 (6)	0.0018 (5)	0.0138 (5)	0.0002 (5)
C14	0.0208 (6)	0.0237 (6)	0.0245 (6)	0.0015 (5)	0.0151 (5)	0.0008 (5)
C15	0.0266 (7)	0.0237 (6)	0.0287 (7)	0.0010 (5)	0.0185 (6)	0.0036 (5)
C16	0.0274 (7)	0.0218 (6)	0.0230 (6)	0.0036 (5)	0.0143 (6)	0.0002 (5)
C17	0.0217 (6)	0.0290 (7)	0.0284 (7)	0.0048 (5)	0.0151 (6)	0.0016 (5)
C18	0.0243 (7)	0.0299 (7)	0.0293 (7)	0.0016 (5)	0.0189 (6)	0.0026 (6)
C19	0.0327 (8)	0.0305 (8)	0.0383 (8)	0.0102 (6)	0.0180 (7)	0.0111 (6)

### (E)-1-[1-(4-tert-Butylphenyl)-2,2-dichloroethenyl]-2-\ (4-methylphenyl)diazene (II) Geometric parameters (Å, º)

**Table:**

Cl1—C2	1.7163 (13)	C10—H10B	0.9800
Cl2—C2	1.7229 (13)	C10—H10C	0.9800
N1—N2	1.2613 (16)	C11—H11A	0.9800
N1—C1	1.4202 (16)	C11—H11B	0.9800
N2—C13	1.4199 (17)	C11—H11C	0.9800
C1—C2	1.3415 (19)	C12—H12A	0.9800
C1—C3	1.4871 (17)	C12—H12B	0.9800
C3—C8	1.3887 (18)	C12—H12C	0.9800
C3—C4	1.3964 (18)	C13—C18	1.3921 (19)
C4—C5	1.3898 (19)	C13—C14	1.3944 (18)
C4—H4	0.9500	C14—C15	1.3844 (19)
C5—C6	1.3942 (18)	C14—H14	0.9500
C5—H5	0.9500	C15—C16	1.394 (2)
C6—C7	1.3995 (18)	C15—H15	0.9500
C6—C9	1.5348 (17)	C16—C17	1.391 (2)
C7—C8	1.3915 (18)	C16—C19	1.5089 (19)
C7—H7	0.9500	C17—C18	1.391 (2)
C8—H8	0.9500	C17—H17	0.9500
C9—C10	1.5303 (18)	C18—H18	0.9500
C9—C11	1.5349 (18)	C19—H19A	0.9800
C9—C12	1.5353 (19)	C19—H19B	0.9800
C10—H10A	0.9800	C19—H19C	0.9800
			
N2—N1—C1	112.90 (11)	H10B—C10—H10C	109.5
N1—N2—C13	113.32 (10)	C9—C11—H11A	109.5
C2—C1—N1	115.67 (11)	C9—C11—H11B	109.5
C2—C1—C3	123.67 (12)	H11A—C11—H11B	109.5
N1—C1—C3	120.60 (11)	C9—C11—H11C	109.5
C1—C2—Cl1	123.05 (10)	H11A—C11—H11C	109.5
C1—C2—Cl2	123.35 (10)	H11B—C11—H11C	109.5
Cl1—C2—Cl2	113.60 (7)	C9—C12—H12A	109.5
C8—C3—C4	118.82 (12)	C9—C12—H12B	109.5
C8—C3—C1	122.23 (12)	H12A—C12—H12B	109.5
C4—C3—C1	118.92 (11)	C9—C12—H12C	109.5
C5—C4—C3	120.59 (12)	H12A—C12—H12C	109.5
C5—C4—H4	119.7	H12B—C12—H12C	109.5
C3—C4—H4	119.7	C18—C13—C14	120.14 (12)
C4—C5—C6	121.38 (12)	C18—C13—N2	117.00 (12)
C4—C5—H5	119.3	C14—C13—N2	122.78 (12)
C6—C5—H5	119.3	C15—C14—C13	119.31 (12)
C5—C6—C7	117.26 (11)	C15—C14—H14	120.3
C5—C6—C9	122.75 (11)	C13—C14—H14	120.3
C7—C6—C9	119.96 (11)	C14—C15—C16	121.31 (13)
C8—C7—C6	121.85 (12)	C14—C15—H15	119.3
C8—C7—H7	119.1	C16—C15—H15	119.3
C6—C7—H7	119.1	C17—C16—C15	118.81 (12)
C3—C8—C7	120.09 (12)	C17—C16—C19	120.69 (13)
C3—C8—H8	120.0	C15—C16—C19	120.49 (13)
C7—C8—H8	120.0	C18—C17—C16	120.58 (13)
C10—C9—C6	112.52 (11)	C18—C17—H17	119.7
C10—C9—C11	107.56 (11)	C16—C17—H17	119.7
C6—C9—C11	109.98 (10)	C17—C18—C13	119.81 (13)
C10—C9—C12	108.35 (12)	C17—C18—H18	120.1
C6—C9—C12	108.31 (10)	C13—C18—H18	120.1
C11—C9—C12	110.09 (12)	C16—C19—H19A	109.5
C9—C10—H10A	109.5	C16—C19—H19B	109.5
C9—C10—H10B	109.5	H19A—C19—H19B	109.5
H10A—C10—H10B	109.5	C16—C19—H19C	109.5
C9—C10—H10C	109.5	H19A—C19—H19C	109.5
H10A—C10—H10C	109.5	H19B—C19—H19C	109.5
			
C1—N1—N2—C13	175.97 (11)	C6—C7—C8—C3	1.1 (2)
N2—N1—C1—C2	164.44 (12)	C5—C6—C9—C10	12.41 (17)
N2—N1—C1—C3	−18.16 (17)	C7—C6—C9—C10	−169.31 (12)
N1—C1—C2—Cl1	179.34 (9)	C5—C6—C9—C11	132.31 (13)
C3—C1—C2—Cl1	2.03 (19)	C7—C6—C9—C11	−49.41 (16)
N1—C1—C2—Cl2	−1.51 (18)	C5—C6—C9—C12	−107.35 (14)
C3—C1—C2—Cl2	−178.82 (10)	C7—C6—C9—C12	70.94 (15)
C2—C1—C3—C8	−65.16 (18)	N1—N2—C13—C18	157.20 (12)
N1—C1—C3—C8	117.65 (14)	N1—N2—C13—C14	−26.07 (18)
C2—C1—C3—C4	116.87 (15)	C18—C13—C14—C15	−0.7 (2)
N1—C1—C3—C4	−60.31 (17)	N2—C13—C14—C15	−177.35 (12)
C8—C3—C4—C5	−0.2 (2)	C13—C14—C15—C16	−0.8 (2)
C1—C3—C4—C5	177.83 (12)	C14—C15—C16—C17	0.7 (2)
C3—C4—C5—C6	0.0 (2)	C14—C15—C16—C19	−179.95 (14)
C4—C5—C6—C7	0.74 (19)	C15—C16—C17—C18	0.9 (2)
C4—C5—C6—C9	179.07 (12)	C19—C16—C17—C18	−178.43 (14)
C5—C6—C7—C8	−1.27 (19)	C16—C17—C18—C13	−2.4 (2)
C9—C6—C7—C8	−179.65 (12)	C14—C13—C18—C17	2.3 (2)
C4—C3—C8—C7	−0.30 (19)	N2—C13—C18—C17	179.13 (12)
C1—C3—C8—C7	−178.27 (12)		

### (E)-1-[1-(4-tert-Butylphenyl)-2,2-dichloroethenyl]-2-\ (4-methylphenyl)diazene (II) Hydrogen-bond geometry (Å, º)

*Cg*1 is the centroid of the 4-*tert*-butylphenyl ring (C3–C8).

**Table:**

*D*—H···*A*	*D*—H	H···*A*	*D*···*A*	*D*—H···*A*
C17—H17···*Cg*1^i^	0.95	2.88	3.675 (2)	142

Symmetry code: (i) −*x*+1/2, *y*+1/2, −*z*+1/2.

### (E)-1-[1-(4-tert-Butylphenyl)-2,2-dichloroethenyl]-2-(4-methoxyphenyl)diazene (III) Crystal data

**Table:**

C_19_H_20_Cl_2_N_2_O	*F*(000) = 760
*M**_r_* = 363.27	*D*_x_ = 1.323 Mg m^−^^3^
Monoclinic, *P*2_1_/*c*	Cu *K*α radiation, λ = 1.54184 Å
*a* = 13.8738 (2) Å	Cell parameters from 17727 reflections
*b* = 12.5946 (2) Å	θ = 3.4–77.6°
*c* = 11.3013 (1) Å	µ = 3.26 mm^−^^1^
β = 112.505 (1)°	*T* = 100 K
*V* = 1824.35 (4) Å^3^	Prism, red
*Z* = 4	0.24 × 0.20 × 0.18 mm

### (E)-1-[1-(4-tert-Butylphenyl)-2,2-dichloroethenyl]-2-(4-methoxyphenyl)diazene (III) Data collection

**Table:**

XtaLAB Synergy, Dualflex, HyPix diffractometer	3603 reflections with *I* > 2σ(*I*)
Radiation source: micro-focus sealed X-ray tube	*R*_int_ = 0.076
φ and ω scans	θ_max_ = 77.9°, θ_min_ = 3.5°
Absorption correction: multi-scan (*CrysAlisPro*; Rigaku OD, 2021)	*h* = −15→17
*T*_min_ = 0.431, *T*_max_ = 0.550	*k* = −15→15
25894 measured reflections	*l* = −14→14
3843 independent reflections	

### (E)-1-[1-(4-tert-Butylphenyl)-2,2-dichloroethenyl]-2-(4-methoxyphenyl)diazene (III) Refinement

**Table:**

Refinement on *F*^2^	0 restraints
Least-squares matrix: full	Hydrogen site location: inferred from neighbouring sites
*R*[*F*^2^ > 2σ(*F*^2^)] = 0.043	H-atom parameters constrained
*wR*(*F*^2^) = 0.118	*w* = 1/[σ^2^(*F*_o_^2^) + (0.0831*P*)^2^ + 0.4008*P*] where *P* = (*F*_o_^2^ + 2*F*_c_^2^)/3
*S* = 1.06	(Δ/σ)_max_ < 0.001
3843 reflections	Δρ_max_ = 0.53 e Å^−^^3^
221 parameters	Δρ_min_ = −0.33 e Å^−^^3^

### (E)-1-[1-(4-tert-Butylphenyl)-2,2-dichloroethenyl]-2-(4-methoxyphenyl)diazene (III) Special details

**Table:**

Geometry. All esds (except the esd in the dihedral angle between two l.s. planes) are estimated using the full covariance matrix. The cell esds are taken into account individually in the estimation of esds in distances, angles and torsion angles; correlations between esds in cell parameters are only used when they are defined by crystal symmetry. An approximate (isotropic) treatment of cell esds is used for estimating esds involving l.s. planes.

### (E)-1-[1-(4-tert-Butylphenyl)-2,2-dichloroethenyl]-2-(4-methoxyphenyl)diazene (III) Fractional atomic coordinates and isotropic or equivalent isotropic displacement parameters (Å^2^)

**Table:**

	*x*	*y*	*z*	*U*_iso_*/*U*_eq_	
C1	0.20324 (11)	0.34882 (11)	0.22693 (13)	0.0187 (3)	
C2	0.14902 (11)	0.30352 (11)	0.11245 (13)	0.0202 (3)	
C3	0.19934 (10)	0.46520 (11)	0.24859 (12)	0.0176 (3)	
C4	0.13412 (10)	0.50501 (11)	0.30620 (13)	0.0189 (3)	
H4	0.093607	0.457391	0.333412	0.023*	
C5	0.12770 (10)	0.61349 (11)	0.32425 (13)	0.0188 (3)	
H5	0.081387	0.638876	0.361713	0.023*	
C6	0.18772 (10)	0.68598 (11)	0.28860 (12)	0.0170 (3)	
C7	0.25514 (11)	0.64453 (12)	0.23454 (14)	0.0213 (3)	
H7	0.298483	0.691647	0.211415	0.026*	
C8	0.26040 (11)	0.53661 (12)	0.21381 (13)	0.0214 (3)	
H8	0.306116	0.511166	0.175511	0.026*	
C9	0.18118 (11)	0.80655 (11)	0.30498 (13)	0.0205 (3)	
C10	0.09974 (13)	0.83569 (12)	0.35970 (16)	0.0268 (3)	
H10A	0.030833	0.811599	0.301035	0.040*	
H10B	0.098759	0.912885	0.370051	0.040*	
H10C	0.117338	0.801296	0.443148	0.040*	
C11	0.28773 (13)	0.84892 (13)	0.39614 (16)	0.0282 (3)	
H11A	0.305696	0.817148	0.481074	0.042*	
H11B	0.284263	0.926299	0.402705	0.042*	
H11C	0.341121	0.830301	0.362727	0.042*	
C12	0.15139 (14)	0.86056 (13)	0.17359 (15)	0.0298 (3)	
H12A	0.205161	0.845923	0.139406	0.045*	
H12B	0.145827	0.937393	0.183169	0.045*	
H12C	0.084214	0.832769	0.114538	0.045*	
C13	0.37438 (10)	0.24915 (11)	0.52562 (13)	0.0184 (3)	
C14	0.39042 (11)	0.14261 (11)	0.50182 (13)	0.0193 (3)	
H14	0.360543	0.115106	0.417218	0.023*	
C15	0.44961 (11)	0.07799 (11)	0.60150 (13)	0.0198 (3)	
H15	0.461097	0.006053	0.585247	0.024*	
C16	0.49311 (10)	0.11785 (11)	0.72709 (13)	0.0186 (3)	
C17	0.47838 (11)	0.22386 (11)	0.75109 (13)	0.0199 (3)	
H17	0.508107	0.251380	0.835695	0.024*	
C18	0.41964 (11)	0.28891 (11)	0.64970 (13)	0.0206 (3)	
H18	0.410262	0.361533	0.665345	0.025*	
C19	0.59249 (13)	0.08155 (13)	0.94780 (14)	0.0275 (3)	
H19A	0.641917	0.139312	0.955751	0.041*	
H19B	0.629032	0.022336	1.003028	0.041*	
H19C	0.536817	0.107057	0.973833	0.041*	
Cl1	0.07489 (3)	0.37510 (3)	−0.01999 (3)	0.02558 (13)	
Cl2	0.14590 (3)	0.16875 (3)	0.08679 (3)	0.02464 (13)	
N1	0.26151 (9)	0.27761 (9)	0.32441 (11)	0.0193 (2)	
N2	0.31434 (9)	0.32201 (9)	0.42989 (11)	0.0195 (2)	
O1	0.54829 (8)	0.04642 (8)	0.81760 (10)	0.0246 (2)	

### (E)-1-[1-(4-tert-Butylphenyl)-2,2-dichloroethenyl]-2-(4-methoxyphenyl)diazene (III) Atomic displacement parameters (Å^2^)

**Table:**

	*U*^11^	*U*^22^	*U*^33^	*U*^12^	*U*^13^	*U*^23^
C1	0.0212 (6)	0.0180 (6)	0.0175 (6)	0.0014 (5)	0.0081 (5)	0.0008 (5)
C2	0.0227 (7)	0.0178 (6)	0.0191 (6)	−0.0003 (5)	0.0070 (5)	0.0001 (5)
C3	0.0202 (6)	0.0168 (6)	0.0139 (6)	0.0012 (5)	0.0042 (5)	0.0002 (5)
C4	0.0200 (6)	0.0193 (7)	0.0168 (6)	−0.0019 (5)	0.0064 (5)	0.0009 (5)
C5	0.0192 (6)	0.0207 (7)	0.0168 (6)	0.0004 (5)	0.0072 (5)	−0.0002 (5)
C6	0.0188 (6)	0.0167 (6)	0.0129 (6)	0.0002 (5)	0.0034 (5)	0.0002 (5)
C7	0.0237 (7)	0.0200 (7)	0.0227 (7)	−0.0028 (5)	0.0116 (6)	0.0008 (5)
C8	0.0240 (7)	0.0217 (7)	0.0219 (6)	0.0017 (5)	0.0126 (5)	0.0008 (5)
C9	0.0253 (7)	0.0156 (6)	0.0193 (6)	−0.0009 (5)	0.0071 (5)	−0.0007 (5)
C10	0.0318 (8)	0.0185 (7)	0.0316 (8)	0.0042 (6)	0.0137 (6)	−0.0015 (6)
C11	0.0293 (8)	0.0225 (7)	0.0301 (8)	−0.0055 (6)	0.0084 (6)	−0.0064 (6)
C12	0.0457 (9)	0.0192 (7)	0.0230 (7)	0.0005 (6)	0.0114 (7)	0.0031 (6)
C13	0.0204 (6)	0.0172 (6)	0.0171 (6)	−0.0002 (5)	0.0065 (5)	0.0010 (5)
C14	0.0219 (7)	0.0182 (6)	0.0179 (6)	−0.0018 (5)	0.0079 (5)	−0.0020 (5)
C15	0.0227 (6)	0.0157 (6)	0.0202 (6)	−0.0001 (5)	0.0073 (5)	−0.0005 (5)
C16	0.0181 (6)	0.0180 (7)	0.0185 (6)	0.0003 (5)	0.0056 (5)	0.0030 (5)
C17	0.0218 (6)	0.0193 (7)	0.0169 (6)	−0.0010 (5)	0.0054 (5)	−0.0012 (5)
C18	0.0242 (7)	0.0165 (6)	0.0198 (6)	−0.0001 (5)	0.0069 (5)	−0.0009 (5)
C19	0.0326 (8)	0.0238 (7)	0.0183 (7)	0.0041 (6)	0.0009 (6)	0.0018 (5)
Cl1	0.0307 (2)	0.0239 (2)	0.01655 (19)	0.00034 (12)	0.00291 (15)	0.00222 (11)
Cl2	0.0306 (2)	0.0173 (2)	0.0225 (2)	−0.00119 (12)	0.00626 (15)	−0.00440 (11)
N1	0.0219 (6)	0.0179 (6)	0.0168 (5)	0.0010 (4)	0.0059 (4)	0.0004 (4)
N2	0.0225 (6)	0.0178 (6)	0.0170 (5)	0.0010 (4)	0.0064 (5)	0.0004 (4)
O1	0.0301 (5)	0.0187 (5)	0.0190 (5)	0.0039 (4)	0.0027 (4)	0.0019 (4)

### (E)-1-[1-(4-tert-Butylphenyl)-2,2-dichloroethenyl]-2-(4-methoxyphenyl)diazene (III) Geometric parameters (Å, º)

**Table:**

C1—C2	1.349 (2)	C11—H11B	0.9800
C1—N1	1.4106 (18)	C11—H11C	0.9800
C1—C3	1.4904 (19)	C12—H12A	0.9800
C2—Cl1	1.7125 (14)	C12—H12B	0.9800
C2—Cl2	1.7196 (15)	C12—H12C	0.9800
C3—C8	1.3913 (19)	C13—C18	1.3920 (19)
C3—C4	1.3947 (19)	C13—C14	1.4029 (19)
C4—C5	1.389 (2)	C13—N2	1.4200 (18)
C4—H4	0.9500	C14—C15	1.377 (2)
C5—C6	1.3951 (19)	C14—H14	0.9500
C5—H5	0.9500	C15—C16	1.405 (2)
C6—C7	1.3993 (19)	C15—H15	0.9500
C6—C9	1.5366 (19)	C16—O1	1.3573 (17)
C7—C8	1.386 (2)	C16—C17	1.393 (2)
C7—H7	0.9500	C17—C18	1.3905 (19)
C8—H8	0.9500	C17—H17	0.9500
C9—C10	1.526 (2)	C18—H18	0.9500
C9—C11	1.538 (2)	C19—O1	1.4305 (18)
C9—C12	1.539 (2)	C19—H19A	0.9800
C10—H10A	0.9800	C19—H19B	0.9800
C10—H10B	0.9800	C19—H19C	0.9800
C10—H10C	0.9800	N1—N2	1.2658 (17)
C11—H11A	0.9800		
			
C2—C1—N1	114.99 (12)	H11A—C11—H11B	109.5
C2—C1—C3	122.10 (13)	C9—C11—H11C	109.5
N1—C1—C3	122.89 (12)	H11A—C11—H11C	109.5
C1—C2—Cl1	122.91 (11)	H11B—C11—H11C	109.5
C1—C2—Cl2	123.23 (11)	C9—C12—H12A	109.5
Cl1—C2—Cl2	113.85 (8)	C9—C12—H12B	109.5
C8—C3—C4	118.26 (13)	H12A—C12—H12B	109.5
C8—C3—C1	121.67 (12)	C9—C12—H12C	109.5
C4—C3—C1	120.07 (12)	H12A—C12—H12C	109.5
C5—C4—C3	120.77 (12)	H12B—C12—H12C	109.5
C5—C4—H4	119.6	C18—C13—C14	119.59 (13)
C3—C4—H4	119.6	C18—C13—N2	116.23 (12)
C4—C5—C6	121.48 (12)	C14—C13—N2	124.19 (12)
C4—C5—H5	119.3	C15—C14—C13	119.80 (13)
C6—C5—H5	119.3	C15—C14—H14	120.1
C5—C6—C7	117.03 (13)	C13—C14—H14	120.1
C5—C6—C9	122.86 (12)	C14—C15—C16	120.40 (13)
C7—C6—C9	120.10 (12)	C14—C15—H15	119.8
C8—C7—C6	121.80 (13)	C16—C15—H15	119.8
C8—C7—H7	119.1	O1—C16—C17	124.82 (13)
C6—C7—H7	119.1	O1—C16—C15	115.13 (12)
C7—C8—C3	120.60 (13)	C17—C16—C15	120.05 (12)
C7—C8—H8	119.7	C18—C17—C16	119.19 (12)
C3—C8—H8	119.7	C18—C17—H17	120.4
C10—C9—C6	111.93 (12)	C16—C17—H17	120.4
C10—C9—C11	108.42 (12)	C17—C18—C13	120.95 (13)
C6—C9—C11	109.73 (12)	C17—C18—H18	119.5
C10—C9—C12	108.52 (13)	C13—C18—H18	119.5
C6—C9—C12	109.09 (11)	O1—C19—H19A	109.5
C11—C9—C12	109.10 (13)	O1—C19—H19B	109.5
C9—C10—H10A	109.5	H19A—C19—H19B	109.5
C9—C10—H10B	109.5	O1—C19—H19C	109.5
H10A—C10—H10B	109.5	H19A—C19—H19C	109.5
C9—C10—H10C	109.5	H19B—C19—H19C	109.5
H10A—C10—H10C	109.5	N2—N1—C1	114.00 (12)
H10B—C10—H10C	109.5	N1—N2—C13	113.04 (11)
C9—C11—H11A	109.5	C16—O1—C19	117.79 (11)
C9—C11—H11B	109.5		
			
N1—C1—C2—Cl1	−178.80 (10)	C7—C6—C9—C11	−62.26 (17)
C3—C1—C2—Cl1	2.57 (19)	C5—C6—C9—C12	−122.02 (15)
N1—C1—C2—Cl2	2.48 (18)	C7—C6—C9—C12	57.21 (17)
C3—C1—C2—Cl2	−176.14 (10)	C18—C13—C14—C15	0.8 (2)
C2—C1—C3—C8	−82.01 (18)	N2—C13—C14—C15	−179.66 (12)
N1—C1—C3—C8	99.47 (16)	C13—C14—C15—C16	0.7 (2)
C2—C1—C3—C4	98.64 (16)	C14—C15—C16—O1	178.87 (12)
N1—C1—C3—C4	−79.88 (17)	C14—C15—C16—C17	−1.4 (2)
C8—C3—C4—C5	2.2 (2)	O1—C16—C17—C18	−179.72 (13)
C1—C3—C4—C5	−178.43 (12)	C15—C16—C17—C18	0.5 (2)
C3—C4—C5—C6	−1.6 (2)	C16—C17—C18—C13	1.0 (2)
C4—C5—C6—C7	−0.37 (19)	C14—C13—C18—C17	−1.7 (2)
C4—C5—C6—C9	178.89 (12)	N2—C13—C18—C17	178.79 (12)
C5—C6—C7—C8	1.7 (2)	C2—C1—N1—N2	177.92 (12)
C9—C6—C7—C8	−177.56 (13)	C3—C1—N1—N2	−3.46 (18)
C6—C7—C8—C3	−1.1 (2)	C1—N1—N2—C13	−178.51 (11)
C4—C3—C8—C7	−0.9 (2)	C18—C13—N2—N1	−168.92 (12)
C1—C3—C8—C7	179.78 (13)	C14—C13—N2—N1	11.54 (18)
C5—C6—C9—C10	−1.92 (18)	C17—C16—O1—C19	1.7 (2)
C7—C6—C9—C10	177.32 (13)	C15—C16—O1—C19	−178.58 (13)
C5—C6—C9—C11	118.51 (14)		

### (E)-1-[1-(4-tert-Butylphenyl)-2,2-dichloroethenyl]-2-(4-methoxyphenyl)diazene (III) Hydrogen-bond geometry (Å, º)

*Cg*1 is the centroid of the 4-*tert*-butylphenyl ring (C3–C8).

**Table:**

*D*—H···*A*	*D*—H	H···*A*	*D*···*A*	*D*—H···*A*
C18—H18···O1^i^	0.95	2.39	3.2753 (17)	155
C19—H19*B*···*Cg*1^ii^	0.98	2.87	3.4276 (17)	117

Symmetry codes: (i) −*x*+1, *y*+1/2, −*z*+3/2; (ii) −*x*+1, *y*−1/2, −*z*+3/2.

### (E)-1-[1-(4-tert-Butylphenyl)-2,2-dichloroethenyl]-2-\ (3-methylphenyl)δiazene (IV) Crystal data

**Table:**

C_19_H_20_Cl_2_N_2_	*Z* = 4
*M**_r_* = 347.27	*F*(000) = 728
Triclinic, *P*1	*D*_x_ = 1.295 Mg m^−^^3^
*a* = 9.8352 (2) Å	Cu *K*α radiation, λ = 1.54184 Å
*b* = 11.8401 (2) Å	Cell parameters from 36250 reflections
*c* = 16.3964 (2) Å	θ = 2.7–77.7°
α = 98.397 (1)°	µ = 3.27 mm^−^^1^
β = 96.189 (1)°	*T* = 100 K
γ = 107.149 (1)°	Prism, red
*V* = 1781.77 (5) Å^3^	0.25 × 0.22 × 0.18 mm

### (E)-1-[1-(4-tert-Butylphenyl)-2,2-dichloroethenyl]-2-\ (3-methylphenyl)δiazene (IV) Data collection

**Table:**

XtaLAB Synergy, Dualflex, HyPix diffractometer	6948 reflections with *I* > 2σ(*I*)
Radiation source: micro-focus sealed X-ray tube	*R*_int_ = 0.071
φ and ω scans	θ_max_ = 77.9°, θ_min_ = 2.8°
Absorption correction: multi-scan (*CrysAlisPro*; Rigaku OD, 2021)	*h* = −12→12
*T*_min_ = 0.328, *T*_max_ = 0.550	*k* = −14→12
53607 measured reflections	*l* = −20→20
7515 independent reflections	

### (E)-1-[1-(4-tert-Butylphenyl)-2,2-dichloroethenyl]-2-\ (3-methylphenyl)δiazene (IV) Refinement

**Table:**

Refinement on *F*^2^	0 restraints
Least-squares matrix: full	Hydrogen site location: inferred from neighbouring sites
*R*[*F*^2^ > 2σ(*F*^2^)] = 0.058	H-atom parameters constrained
*wR*(*F*^2^) = 0.171	*w* = 1/[σ^2^(*F*_o_^2^) + (0.1247*P*)^2^ + 0.6991*P*] where *P* = (*F*_o_^2^ + 2*F*_c_^2^)/3
*S* = 1.04	(Δ/σ)_max_ = 0.001
7515 reflections	Δρ_max_ = 0.93 e Å^−^^3^
423 parameters	Δρ_min_ = −0.63 e Å^−^^3^

### (E)-1-[1-(4-tert-Butylphenyl)-2,2-dichloroethenyl]-2-\ (3-methylphenyl)δiazene (IV) Special details

**Table:**

Geometry. All esds (except the esd in the dihedral angle between two l.s. planes) are estimated using the full covariance matrix. The cell esds are taken into account individually in the estimation of esds in distances, angles and torsion angles; correlations between esds in cell parameters are only used when they are defined by crystal symmetry. An approximate (isotropic) treatment of cell esds is used for estimating esds involving l.s. planes.

### (E)-1-[1-(4-tert-Butylphenyl)-2,2-dichloroethenyl]-2-\ (3-methylphenyl)δiazene (IV) Fractional atomic coordinates and isotropic or equivalent isotropic displacement parameters (Å^2^)

**Table:**

	*x*	*y*	*z*	*U*_iso_*/*U*_eq_	
C1	0.61178 (18)	0.35073 (16)	0.16342 (12)	0.0249 (4)	
C2	0.6208 (2)	0.27286 (17)	0.09726 (12)	0.0286 (4)	
C3	0.58777 (18)	0.31800 (15)	0.24597 (11)	0.0230 (3)	
C4	0.46666 (19)	0.32673 (16)	0.28085 (12)	0.0265 (4)	
H4	0.393723	0.347551	0.249429	0.032*	
C5	0.45226 (19)	0.30530 (16)	0.36066 (12)	0.0265 (4)	
H5	0.369067	0.311600	0.383040	0.032*	
C6	0.55770 (18)	0.27437 (15)	0.40967 (11)	0.0239 (3)	
C7	0.67636 (19)	0.26365 (16)	0.37312 (12)	0.0255 (4)	
H7	0.748785	0.241541	0.403958	0.031*	
C8	0.69106 (18)	0.28451 (16)	0.29288 (12)	0.0255 (4)	
H8	0.772654	0.275817	0.269671	0.031*	
C9	0.5398 (2)	0.25476 (16)	0.49849 (12)	0.0272 (4)	
C10	0.5205 (2)	0.36724 (18)	0.54994 (13)	0.0333 (4)	
H10A	0.603703	0.437581	0.550108	0.050*	
H10B	0.512989	0.355418	0.607413	0.050*	
H10C	0.432478	0.380217	0.525009	0.050*	
C11	0.4060 (2)	0.14560 (18)	0.49507 (13)	0.0344 (4)	
H11A	0.320435	0.160683	0.468623	0.052*	
H11B	0.394079	0.132610	0.551906	0.052*	
H11C	0.418147	0.073943	0.462518	0.052*	
C12	0.6708 (2)	0.2312 (2)	0.54356 (13)	0.0343 (4)	
H12A	0.681949	0.157211	0.513836	0.051*	
H12B	0.656545	0.222374	0.600823	0.051*	
H12C	0.757630	0.298939	0.544841	0.051*	
C13	0.67080 (18)	0.66543 (17)	0.20121 (12)	0.0267 (4)	
C14	0.67993 (19)	0.69894 (17)	0.12348 (12)	0.0273 (4)	
H14	0.659272	0.638881	0.074597	0.033*	
C15	0.7192 (2)	0.81996 (18)	0.11699 (13)	0.0300 (4)	
C16	0.7485 (2)	0.90601 (17)	0.18984 (13)	0.0313 (4)	
H16	0.776188	0.988838	0.186220	0.038*	
C17	0.7382 (2)	0.87368 (18)	0.26740 (13)	0.0313 (4)	
H17	0.757830	0.933887	0.316095	0.038*	
C18	0.69929 (19)	0.75318 (17)	0.27368 (13)	0.0284 (4)	
H18	0.691972	0.730266	0.326601	0.034*	
C19	0.7313 (3)	0.85617 (19)	0.03339 (14)	0.0372 (5)	
H19A	0.681665	0.915895	0.027531	0.056*	
H19B	0.686864	0.785258	−0.010902	0.056*	
H19C	0.833165	0.891001	0.028971	0.056*	
C20	0.91894 (19)	0.62019 (16)	0.82034 (12)	0.0257 (4)	
C21	0.9535 (2)	0.69058 (16)	0.89645 (12)	0.0290 (4)	
C22	0.87259 (19)	0.48653 (16)	0.80936 (11)	0.0237 (3)	
C23	0.72883 (19)	0.41702 (17)	0.79251 (12)	0.0278 (4)	
H23	0.656334	0.454890	0.787674	0.033*	
C24	0.68937 (18)	0.29242 (16)	0.78259 (12)	0.0261 (4)	
H24	0.589993	0.246660	0.771607	0.031*	
C25	0.79213 (18)	0.23299 (15)	0.78837 (11)	0.0228 (3)	
C26	0.93658 (19)	0.30439 (17)	0.80626 (12)	0.0286 (4)	
H26	1.009196	0.266638	0.811020	0.034*	
C27	0.97708 (19)	0.42862 (17)	0.81727 (13)	0.0292 (4)	
H27	1.076283	0.474732	0.830261	0.035*	
C28	0.75234 (19)	0.09595 (16)	0.77649 (11)	0.0253 (4)	
C29	0.5908 (2)	0.03328 (16)	0.74827 (13)	0.0306 (4)	
H29A	0.537672	0.059455	0.790546	0.046*	
H29B	0.570431	−0.054050	0.741081	0.046*	
H29C	0.560719	0.054255	0.695097	0.046*	
C30	0.8320 (2)	0.05048 (18)	0.70974 (14)	0.0341 (4)	
H30A	0.808999	0.077558	0.657793	0.051*	
H30B	0.801615	−0.037641	0.699604	0.051*	
H30C	0.936143	0.082550	0.729276	0.051*	
C31	0.7981 (2)	0.06146 (18)	0.85985 (14)	0.0369 (5)	
H31A	0.902082	0.099535	0.877774	0.055*	
H31B	0.774929	−0.026150	0.852401	0.055*	
H31C	0.746315	0.089054	0.902386	0.055*	
C32	0.92807 (19)	0.68230 (17)	0.61640 (12)	0.0254 (4)	
C33	0.90203 (19)	0.61415 (17)	0.53664 (12)	0.0279 (4)	
H33	0.868253	0.528857	0.529137	0.034*	
C34	0.9247 (2)	0.66907 (18)	0.46696 (12)	0.0293 (4)	
C35	0.9724 (2)	0.79377 (18)	0.48010 (13)	0.0310 (4)	
H35	0.988008	0.832852	0.433702	0.037*	
C36	0.9977 (2)	0.86290 (18)	0.55985 (13)	0.0328 (4)	
H36	1.029797	0.948185	0.567020	0.039*	
C37	0.9767 (2)	0.80869 (17)	0.62886 (12)	0.0293 (4)	
H37	0.994786	0.855806	0.683334	0.035*	
C38	0.8987 (3)	0.5940 (2)	0.38145 (13)	0.0393 (5)	
H38A	0.955999	0.538846	0.381276	0.059*	
H38B	0.926960	0.646511	0.341058	0.059*	
H38C	0.796204	0.547539	0.366089	0.059*	
Cl1	0.58942 (6)	0.12256 (4)	0.09905 (3)	0.03558 (15)	
Cl2	0.66274 (6)	0.31202 (5)	0.00425 (3)	0.03661 (15)	
Cl3	0.93882 (6)	0.63467 (4)	0.98652 (3)	0.03543 (15)	
Cl4	1.01859 (6)	0.84553 (4)	0.91208 (3)	0.03931 (16)	
N1	0.63865 (16)	0.47049 (14)	0.15050 (10)	0.0266 (3)	
N2	0.63619 (16)	0.54441 (14)	0.21407 (10)	0.0260 (3)	
N3	0.93773 (17)	0.68198 (14)	0.75331 (10)	0.0269 (3)	
N4	0.90357 (16)	0.61543 (14)	0.68230 (10)	0.0264 (3)	

### (E)-1-[1-(4-tert-Butylphenyl)-2,2-dichloroethenyl]-2-\ (3-methylphenyl)δiazene (IV) Atomic displacement parameters (Å^2^)

**Table:**

	*U*^11^	*U*^22^	*U*^33^	*U*^12^	*U*^13^	*U*^23^
C1	0.0242 (8)	0.0224 (8)	0.0308 (9)	0.0099 (6)	0.0048 (6)	0.0082 (7)
C2	0.0314 (9)	0.0264 (9)	0.0314 (9)	0.0123 (7)	0.0071 (7)	0.0083 (7)
C3	0.0257 (8)	0.0165 (7)	0.0286 (9)	0.0091 (6)	0.0043 (6)	0.0050 (6)
C4	0.0261 (8)	0.0235 (9)	0.0346 (9)	0.0135 (7)	0.0045 (7)	0.0085 (7)
C5	0.0254 (8)	0.0246 (9)	0.0339 (10)	0.0126 (7)	0.0073 (7)	0.0075 (7)
C6	0.0260 (8)	0.0180 (8)	0.0289 (9)	0.0088 (6)	0.0043 (7)	0.0047 (6)
C7	0.0256 (8)	0.0229 (8)	0.0308 (9)	0.0113 (6)	0.0035 (7)	0.0075 (7)
C8	0.0232 (8)	0.0231 (8)	0.0339 (9)	0.0116 (6)	0.0058 (7)	0.0067 (7)
C9	0.0302 (9)	0.0251 (9)	0.0286 (9)	0.0109 (7)	0.0061 (7)	0.0065 (7)
C10	0.0399 (10)	0.0312 (10)	0.0331 (10)	0.0164 (8)	0.0101 (8)	0.0048 (8)
C11	0.0365 (10)	0.0300 (10)	0.0355 (10)	0.0056 (8)	0.0084 (8)	0.0102 (8)
C12	0.0386 (10)	0.0387 (11)	0.0311 (10)	0.0184 (8)	0.0056 (8)	0.0107 (8)
C13	0.0223 (8)	0.0256 (9)	0.0354 (10)	0.0103 (7)	0.0055 (7)	0.0093 (7)
C14	0.0268 (8)	0.0248 (9)	0.0329 (9)	0.0121 (7)	0.0042 (7)	0.0057 (7)
C15	0.0276 (8)	0.0309 (10)	0.0346 (10)	0.0117 (7)	0.0048 (7)	0.0109 (8)
C16	0.0284 (9)	0.0236 (9)	0.0430 (11)	0.0100 (7)	0.0040 (8)	0.0075 (8)
C17	0.0306 (9)	0.0262 (9)	0.0367 (10)	0.0113 (7)	0.0027 (7)	0.0013 (7)
C18	0.0267 (8)	0.0266 (9)	0.0344 (10)	0.0118 (7)	0.0044 (7)	0.0064 (7)
C19	0.0501 (12)	0.0270 (10)	0.0386 (11)	0.0146 (9)	0.0089 (9)	0.0129 (8)
C20	0.0255 (8)	0.0235 (9)	0.0303 (9)	0.0093 (7)	0.0070 (7)	0.0070 (7)
C21	0.0361 (9)	0.0206 (8)	0.0327 (10)	0.0097 (7)	0.0094 (7)	0.0082 (7)
C22	0.0280 (8)	0.0214 (8)	0.0237 (8)	0.0101 (7)	0.0059 (6)	0.0048 (6)
C23	0.0270 (8)	0.0233 (9)	0.0364 (10)	0.0137 (7)	0.0045 (7)	0.0048 (7)
C24	0.0214 (8)	0.0232 (9)	0.0344 (9)	0.0086 (6)	0.0033 (7)	0.0053 (7)
C25	0.0269 (8)	0.0221 (8)	0.0224 (8)	0.0117 (7)	0.0042 (6)	0.0048 (6)
C26	0.0254 (8)	0.0271 (9)	0.0371 (10)	0.0145 (7)	0.0039 (7)	0.0056 (7)
C27	0.0217 (8)	0.0260 (9)	0.0398 (10)	0.0076 (7)	0.0048 (7)	0.0059 (7)
C28	0.0287 (8)	0.0212 (8)	0.0287 (9)	0.0120 (7)	0.0035 (7)	0.0052 (6)
C29	0.0303 (9)	0.0197 (8)	0.0417 (11)	0.0071 (7)	0.0063 (8)	0.0059 (7)
C30	0.0329 (9)	0.0263 (9)	0.0426 (11)	0.0127 (7)	0.0069 (8)	−0.0026 (8)
C31	0.0492 (12)	0.0264 (10)	0.0375 (11)	0.0158 (8)	−0.0009 (9)	0.0112 (8)
C32	0.0246 (8)	0.0269 (9)	0.0293 (9)	0.0130 (7)	0.0054 (7)	0.0086 (7)
C33	0.0278 (8)	0.0237 (9)	0.0336 (10)	0.0113 (7)	0.0020 (7)	0.0050 (7)
C34	0.0298 (9)	0.0309 (10)	0.0300 (9)	0.0159 (7)	0.0008 (7)	0.0041 (7)
C35	0.0352 (9)	0.0308 (10)	0.0324 (10)	0.0168 (8)	0.0052 (7)	0.0100 (7)
C36	0.0411 (10)	0.0254 (9)	0.0351 (10)	0.0147 (8)	0.0052 (8)	0.0078 (8)
C37	0.0340 (9)	0.0263 (9)	0.0315 (9)	0.0154 (7)	0.0052 (7)	0.0053 (7)
C38	0.0563 (13)	0.0344 (11)	0.0305 (10)	0.0231 (10)	−0.0010 (9)	0.0040 (8)
Cl1	0.0505 (3)	0.0238 (3)	0.0367 (3)	0.0175 (2)	0.0099 (2)	0.00496 (19)
Cl2	0.0483 (3)	0.0372 (3)	0.0302 (3)	0.0181 (2)	0.0131 (2)	0.01026 (19)
Cl3	0.0526 (3)	0.0272 (3)	0.0286 (3)	0.0135 (2)	0.0108 (2)	0.00704 (18)
Cl4	0.0583 (3)	0.0196 (2)	0.0377 (3)	0.0078 (2)	0.0127 (2)	0.00383 (18)
N1	0.0252 (7)	0.0249 (8)	0.0330 (8)	0.0104 (6)	0.0055 (6)	0.0099 (6)
N2	0.0246 (7)	0.0228 (7)	0.0335 (8)	0.0108 (6)	0.0052 (6)	0.0072 (6)
N3	0.0290 (7)	0.0252 (8)	0.0299 (8)	0.0114 (6)	0.0074 (6)	0.0079 (6)
N4	0.0253 (7)	0.0259 (8)	0.0311 (8)	0.0116 (6)	0.0044 (6)	0.0079 (6)

### (E)-1-[1-(4-tert-Butylphenyl)-2,2-dichloroethenyl]-2-\ (3-methylphenyl)δiazene (IV) Geometric parameters (Å, º)

**Table:**

C1—C2	1.346 (3)	C20—N3	1.406 (2)
C1—N1	1.416 (2)	C20—C22	1.489 (2)
C1—C3	1.485 (2)	C21—Cl3	1.7074 (19)
C2—Cl2	1.715 (2)	C21—Cl4	1.7252 (19)
C2—Cl1	1.7193 (19)	C22—C23	1.384 (3)
C3—C8	1.392 (2)	C22—C27	1.399 (2)
C3—C4	1.400 (2)	C23—C24	1.390 (3)
C4—C5	1.383 (3)	C23—H23	0.9500
C4—H4	0.9500	C24—C25	1.394 (2)
C5—C6	1.409 (2)	C24—H24	0.9500
C5—H5	0.9500	C25—C26	1.396 (3)
C6—C7	1.398 (2)	C25—C28	1.530 (2)
C6—C9	1.528 (2)	C26—C27	1.384 (3)
C7—C8	1.389 (3)	C26—H26	0.9500
C7—H7	0.9500	C27—H27	0.9500
C8—H8	0.9500	C28—C29	1.528 (2)
C9—C12	1.533 (3)	C28—C30	1.537 (3)
C9—C11	1.538 (3)	C28—C31	1.541 (3)
C9—C10	1.542 (3)	C29—H29A	0.9800
C10—H10A	0.9800	C29—H29B	0.9800
C10—H10B	0.9800	C29—H29C	0.9800
C10—H10C	0.9800	C30—H30A	0.9800
C11—H11A	0.9800	C30—H30B	0.9800
C11—H11B	0.9800	C30—H30C	0.9800
C11—H11C	0.9800	C31—H31A	0.9800
C12—H12A	0.9800	C31—H31B	0.9800
C12—H12B	0.9800	C31—H31C	0.9800
C12—H12C	0.9800	C32—C33	1.387 (3)
C13—C14	1.393 (3)	C32—C37	1.406 (3)
C13—C18	1.402 (3)	C32—N4	1.430 (2)
C13—N2	1.425 (2)	C33—C34	1.402 (3)
C14—C15	1.393 (3)	C33—H33	0.9500
C14—H14	0.9500	C34—C35	1.387 (3)
C15—C16	1.395 (3)	C34—C38	1.499 (3)
C15—C19	1.501 (3)	C35—C36	1.392 (3)
C16—C17	1.386 (3)	C35—H35	0.9500
C16—H16	0.9500	C36—C37	1.385 (3)
C17—C18	1.386 (3)	C36—H36	0.9500
C17—H17	0.9500	C37—H37	0.9500
C18—H18	0.9500	C38—H38A	0.9800
C19—H19A	0.9800	C38—H38B	0.9800
C19—H19B	0.9800	C38—H38C	0.9800
C19—H19C	0.9800	N1—N2	1.267 (2)
C20—C21	1.344 (3)	N3—N4	1.257 (2)
			
C2—C1—N1	114.51 (16)	N3—C20—C22	123.14 (16)
C2—C1—C3	123.57 (16)	C20—C21—Cl3	123.03 (15)
N1—C1—C3	121.73 (16)	C20—C21—Cl4	123.05 (15)
C1—C2—Cl2	124.11 (15)	Cl3—C21—Cl4	113.91 (11)
C1—C2—Cl1	122.10 (15)	C23—C22—C27	118.47 (16)
Cl2—C2—Cl1	113.79 (11)	C23—C22—C20	122.24 (16)
C8—C3—C4	118.29 (17)	C27—C22—C20	119.28 (16)
C8—C3—C1	120.08 (15)	C22—C23—C24	120.73 (16)
C4—C3—C1	121.52 (15)	C22—C23—H23	119.6
C5—C4—C3	120.57 (16)	C24—C23—H23	119.6
C5—C4—H4	119.7	C23—C24—C25	121.58 (16)
C3—C4—H4	119.7	C23—C24—H24	119.2
C4—C5—C6	121.76 (16)	C25—C24—H24	119.2
C4—C5—H5	119.1	C24—C25—C26	117.00 (16)
C6—C5—H5	119.1	C24—C25—C28	122.87 (15)
C7—C6—C5	116.83 (16)	C26—C25—C28	120.13 (15)
C7—C6—C9	123.00 (15)	C27—C26—C25	121.91 (16)
C5—C6—C9	120.17 (16)	C27—C26—H26	119.0
C8—C7—C6	121.65 (16)	C25—C26—H26	119.0
C8—C7—H7	119.2	C26—C27—C22	120.28 (16)
C6—C7—H7	119.2	C26—C27—H27	119.9
C7—C8—C3	120.87 (16)	C22—C27—H27	119.9
C7—C8—H8	119.6	C29—C28—C25	112.29 (14)
C3—C8—H8	119.6	C29—C28—C30	107.92 (15)
C6—C9—C12	112.17 (15)	C25—C28—C30	109.58 (15)
C6—C9—C11	109.16 (15)	C29—C28—C31	108.82 (16)
C12—C9—C11	108.39 (16)	C25—C28—C31	109.01 (15)
C6—C9—C10	109.85 (15)	C30—C28—C31	109.17 (16)
C12—C9—C10	107.88 (16)	C28—C29—H29A	109.5
C11—C9—C10	109.34 (16)	C28—C29—H29B	109.5
C9—C10—H10A	109.5	H29A—C29—H29B	109.5
C9—C10—H10B	109.5	C28—C29—H29C	109.5
H10A—C10—H10B	109.5	H29A—C29—H29C	109.5
C9—C10—H10C	109.5	H29B—C29—H29C	109.5
H10A—C10—H10C	109.5	C28—C30—H30A	109.5
H10B—C10—H10C	109.5	C28—C30—H30B	109.5
C9—C11—H11A	109.5	H30A—C30—H30B	109.5
C9—C11—H11B	109.5	C28—C30—H30C	109.5
H11A—C11—H11B	109.5	H30A—C30—H30C	109.5
C9—C11—H11C	109.5	H30B—C30—H30C	109.5
H11A—C11—H11C	109.5	C28—C31—H31A	109.5
H11B—C11—H11C	109.5	C28—C31—H31B	109.5
C9—C12—H12A	109.5	H31A—C31—H31B	109.5
C9—C12—H12B	109.5	C28—C31—H31C	109.5
H12A—C12—H12B	109.5	H31A—C31—H31C	109.5
C9—C12—H12C	109.5	H31B—C31—H31C	109.5
H12A—C12—H12C	109.5	C33—C32—C37	120.41 (17)
H12B—C12—H12C	109.5	C33—C32—N4	115.61 (16)
C14—C13—C18	120.31 (17)	C37—C32—N4	123.98 (17)
C14—C13—N2	124.27 (17)	C32—C33—C34	121.09 (17)
C18—C13—N2	115.41 (17)	C32—C33—H33	119.5
C15—C14—C13	120.41 (18)	C34—C33—H33	119.5
C15—C14—H14	119.8	C35—C34—C33	117.91 (18)
C13—C14—H14	119.8	C35—C34—C38	121.73 (18)
C14—C15—C16	118.46 (18)	C33—C34—C38	120.36 (18)
C14—C15—C19	120.42 (18)	C34—C35—C36	121.39 (18)
C16—C15—C19	121.12 (18)	C34—C35—H35	119.3
C17—C16—C15	121.63 (18)	C36—C35—H35	119.3
C17—C16—H16	119.2	C37—C36—C35	120.73 (18)
C15—C16—H16	119.2	C37—C36—H36	119.6
C16—C17—C18	119.78 (18)	C35—C36—H36	119.6
C16—C17—H17	120.1	C36—C37—C32	118.46 (18)
C18—C17—H17	120.1	C36—C37—H37	120.8
C17—C18—C13	119.40 (18)	C32—C37—H37	120.8
C17—C18—H18	120.3	C34—C38—H38A	109.5
C13—C18—H18	120.3	C34—C38—H38B	109.5
C15—C19—H19A	109.5	H38A—C38—H38B	109.5
C15—C19—H19B	109.5	C34—C38—H38C	109.5
H19A—C19—H19B	109.5	H38A—C38—H38C	109.5
C15—C19—H19C	109.5	H38B—C38—H38C	109.5
H19A—C19—H19C	109.5	N2—N1—C1	114.26 (15)
H19B—C19—H19C	109.5	N1—N2—C13	113.47 (16)
C21—C20—N3	115.13 (16)	N4—N3—C20	114.67 (16)
C21—C20—C22	121.64 (16)	N3—N4—C32	112.53 (15)
			
N1—C1—C2—Cl2	0.6 (2)	N3—C20—C22—C23	−86.1 (2)
C3—C1—C2—Cl2	−174.56 (13)	C21—C20—C22—C27	−81.6 (2)
N1—C1—C2—Cl1	−178.98 (13)	N3—C20—C22—C27	94.7 (2)
C3—C1—C2—Cl1	5.9 (3)	C27—C22—C23—C24	−0.8 (3)
C2—C1—C3—C8	64.6 (2)	C20—C22—C23—C24	179.97 (17)
N1—C1—C3—C8	−110.21 (19)	C22—C23—C24—C25	−0.6 (3)
C2—C1—C3—C4	−119.3 (2)	C23—C24—C25—C26	1.3 (3)
N1—C1—C3—C4	65.9 (2)	C23—C24—C25—C28	−179.07 (17)
C8—C3—C4—C5	1.5 (3)	C24—C25—C26—C27	−0.5 (3)
C1—C3—C4—C5	−174.67 (16)	C28—C25—C26—C27	179.86 (17)
C3—C4—C5—C6	0.1 (3)	C25—C26—C27—C22	−1.0 (3)
C4—C5—C6—C7	−1.4 (3)	C23—C22—C27—C26	1.6 (3)
C4—C5—C6—C9	178.32 (16)	C20—C22—C27—C26	−179.16 (17)
C5—C6—C7—C8	1.1 (3)	C24—C25—C28—C29	6.5 (2)
C9—C6—C7—C8	−178.62 (16)	C26—C25—C28—C29	−173.87 (17)
C6—C7—C8—C3	0.5 (3)	C24—C25—C28—C30	126.39 (19)
C4—C3—C8—C7	−1.8 (3)	C26—C25—C28—C30	−54.0 (2)
C1—C3—C8—C7	174.42 (16)	C24—C25—C28—C31	−114.2 (2)
C7—C6—C9—C12	4.0 (2)	C26—C25—C28—C31	65.5 (2)
C5—C6—C9—C12	−175.72 (17)	C37—C32—C33—C34	0.6 (3)
C7—C6—C9—C11	−116.16 (19)	N4—C32—C33—C34	−178.73 (16)
C5—C6—C9—C11	64.1 (2)	C32—C33—C34—C35	−0.8 (3)
C7—C6—C9—C10	123.96 (18)	C32—C33—C34—C38	178.83 (18)
C5—C6—C9—C10	−55.7 (2)	C33—C34—C35—C36	0.3 (3)
C18—C13—C14—C15	0.8 (3)	C38—C34—C35—C36	−179.32 (19)
N2—C13—C14—C15	−177.87 (16)	C34—C35—C36—C37	0.4 (3)
C13—C14—C15—C16	−0.2 (3)	C35—C36—C37—C32	−0.5 (3)
C13—C14—C15—C19	179.04 (17)	C33—C32—C37—C36	0.0 (3)
C14—C15—C16—C17	−0.6 (3)	N4—C32—C37—C36	179.35 (17)
C19—C15—C16—C17	−179.74 (18)	C2—C1—N1—N2	−177.09 (16)
C15—C16—C17—C18	0.6 (3)	C3—C1—N1—N2	−1.8 (2)
C16—C17—C18—C13	0.0 (3)	C1—N1—N2—C13	176.92 (14)
C14—C13—C18—C17	−0.7 (3)	C14—C13—N2—N1	11.6 (2)
N2—C13—C18—C17	178.04 (16)	C18—C13—N2—N1	−167.08 (15)
N3—C20—C21—Cl3	179.92 (13)	C21—C20—N3—N4	−179.22 (16)
C22—C20—C21—Cl3	−3.5 (3)	C22—C20—N3—N4	4.3 (2)
N3—C20—C21—Cl4	−0.8 (2)	C20—N3—N4—C32	−178.50 (14)
C22—C20—C21—Cl4	175.73 (13)	C33—C32—N4—N3	175.76 (15)
C21—C20—C22—C23	97.6 (2)	C37—C32—N4—N3	−3.6 (2)

### (E)-1-[1-(4-tert-Butylphenyl)-2,2-dichloroethenyl]-2-\ (3-methylphenyl)δiazene (IV) Hydrogen-bond geometry (Å, º)

*Cg*1 and *Cg*2 are the centroids of the 4-*tert*-butylphenyl rings [(IVA: C3–C8 and (IVB): C22–C27, respectively]. *Cg*4 is the centroid of the 3-methylphenyl ring (C32–C37) of molecule (IVB).

**Table:**

*D*—H···*A*	*D*—H	H···*A*	*D*···*A*	*D*—H···*A*
C7—H7···*Cg*4^i^	0.95	2.91	3.768 (2)	151
C24—H24···*Cg*2^ii^	0.95	2.97	3.824 (2)	150
C29—H29*B*···*Cg*1^iii^	0.98	2.78	3.706 (2)	157

Symmetry codes: (i) −*x*+2, −*y*+1, −*z*+1; (ii) −*x*+1, −*y*+1, −*z*+1; (iii) −*x*+1, −*y*, −*z*+1.
